# Multinomial logistic regression approach to haplotype association analysis in population-based case-control studies

**DOI:** 10.1186/1471-2156-7-43

**Published:** 2006-08-15

**Authors:** Yi-Hau Chen, Jau-Tsuen Kao

**Affiliations:** 1 Institute of Statistical Science, Academia Sinica, Taipei 11529, Taiwan; 2 Department of Clinical Laboratory Sciences and Medical Biotechnology, College of Medicine, National Taiwan University, Taipei, Taiwan

## Abstract

**Background:**

The genetic association analysis using haplotypes as basic genetic units is anticipated to be a powerful strategy towards the discovery of genes predisposing human complex diseases. In particular, the increasing availability of high-resolution genetic markers such as the single-nucleotide polymorphisms (SNPs) has made haplotype-based association analysis an attractive alternative to single marker analysis.

**Results:**

We consider haplotype association analysis under the population-based case-control study design. A multinomial logistic model is proposed for haplotype analysis with unphased genotype data, which can be decomposed into a prospective logistic model for disease risk as well as a model for the haplotype-pair distribution in the control population. Environmental factors can be readily incorporated and hence the haplotype-environment interaction can be assessed in the proposed model. The maximum likelihood estimation with unphased genotype data can be conveniently implemented in the proposed model by applying the EM algorithm to a prospective multinomial logistic regression model and ignoring the case-control design. We apply the proposed method to the hypertriglyceridemia study and identifies 3 haplotypes in the apolipoprotein A5 gene that are associated with increased risk for hypertriglyceridemia. A haplotype-age interaction effect is also identified. Simulation studies show that the proposed estimator has satisfactory finite-sample performances.

**Conclusion:**

Our results suggest that the proposed method can serve as a useful alternative to existing methods and a reliable tool for the case-control haplotype-based association analysis.

## Background

Genetic association analysis aims to detect gene-disease association through linkage-disequilibrium of a disease susceptibility gene with adjacent genetic markers. Historically, association analysis was limited to single markers. It is anticipated that greater power may be gained by utilizing linkage-disequilibrium information from multiple markers simultaneously. This anticipation, together with recent advances of the availability of high-resolution genetic markers, in particular the single-nucleotide polymorphisms (SNPs), has motivated the use of haplotypes, which are specific combinations of closely linked genetic markers on a chromosome, as the basic genetic units for association analysis. In addition, the biological advantage for haplotype-based analysis is that it can directly identify unique chromosomal segments that contain disease susceptibility genes by assessing the haplotype-specific risk for disease. Schaid [1] provided a detailed and excellent review for haplotype-based association analysis.

The population-based case-control study design has been popular for genetic association analysis due to its cost-efficiency in collecting the data. If the haplotype pair for each individual is directly observable, traditional logistic regression analysis can be applied to assess the haplotype-disease association, possibly adjusting for environmental/demographical factors, and to evaluate the haplotype-environment interactions. According to Prentice and Pyke [2], with case-control data, maximum likelihood estimation of the association (odds-ratio) parameters in logistic regression model can be simply carried out by fitting a prospective logistic model and ignoring the case-control design. Note that, in traditional logistic regression analysis, no modeling assumptions are made on the distribution of the covariates (haplotype and/or environmental factors), that is, the covariate distribution is treated fully nonparametrically.

Usually, the haplotype information is not directly observed and is subject to ambiguity because we can only observe the "unphased" genotype data where the "phase information", i.e., the arrangement of alleles on each of the two chromosomes, is unavailable. There has been rich literature of haplotype inference in general populations, see for example the EM algorithms by Excoffier and Slatkin [3] and Li et al. [4], and the Bayesian methods in Stephens et al. [5] and Niu et al. [6]. To recover the phase information and to ensure the identifiability of the association parameters, in general we need to impose some modeling assumptions on the distribution of haplotype pairs; see Epstein and Satten [7] for related issues when no environmental covariates are considered. One common assumption for such a model is Hardy-Weinberg equilibrium (HWE) in the general population. When environmental covariates are considered, further assumptions regarding relationship between haplotype pairs and environmental factors may be required. One convenient and generally reasonable assumption for this is the haplotype-environment independence [8,9], which assumes that a subject's haplotype-pair features are independent of his/her environmental exposures.

In this work, to assess the haplotype-environment associations with disease phenotype, we will first propose a novel modeling setup that is based on a multinomial logistic regression model, where the various combinations of disease and haplotype-pair categories are treated as multinomial outcomes, and the environmental/demographical factors are used as covariates. We show that the proposed multinomial logistic model can be decomposed into a prospective logistic disease model relating the haplotype and environmental factors with disease, as well as a parametric model for the haplotype-pair distribution, conditional on the environmental covariates, among the control population. Compared to some existing methods such as the one proposed by Spinka et al. [9], our proposal differs from theirs in the way to model the haplotype-pair distribution: In our proposal the model for the haplotype-pair distribution is specified only for the control population, while in the method of Spinka et al. such a model is specified for the whole population. Epstein and Satten [7] uses the same modeling strategy as ours, but their proposal cannot allow for environmental covariates. Our proposal is equivalent to the method of Epstein and Satten in the absence of environmental covariates and extends their method to incorporate environmental covariates.

The major advantage of the proposed approach lies in its computational convenience; it can be simply implemented by an iterative reweighted fit of a multinomial logistic regression model. Note that, when the model for the haplotype-pair distribution is specified for the whole population as in the method of Spinka et al. [9], the true intercept parameter in the prospective disease model, which quantifies the baseline population disease risk, appears as a separate parameter and needs to be estimated. Since usually there is little information on this parameter in a case-control sample, Spinka et al. [9] suggested using external information on the population disease prevalence or the rare-disease approximation to avoid estimating the true intercept parameter. In contrast, in our proposal the true intercept parameter does not appear as a separate parameter and hence needs not be estimated even when the disease is common and the population disease prevalence is unknown. Hence the proposed method has wide applicability. Simulation results reveal that the finite sample properties of the proposed estimates are satisfactory.

## Methods

### Model

Let *D *denote the disease phenotype with *D *= 1 indicating disease and *D *= 0 indicating no disease, *G *the unphased multilocus genotype for a series tightly linked SNPs, and *X *the demographical/environmental covariates. Suppose that there are *J *possible haplotypes denoted by {*ħ*_0_,..., *ħ*_*J*-1_} with *ħ*_0 _indicating the "reference" haplotype (usually the most frequent haplotype). Let (*H*^1^, *H*^2^) be the haplotype pair (diplotype) for a subject and label all possible haplotype pairs by *H *= 0, 1, ..., *M *- 1 so that "0" represents the reference haplotype pair (*ħ*_0_, *ħ*_0_) and *M *is the number of all possible haplotype pairs in the sample.

The multinomial logistic model is a useful tool for regression analysis with multinomial responses [10,11]. Considering the various combinations of (*D*, *H*) as multinomial responses, we propose to base the haplotype association analysis on the following multinomial logistic model:

log⁡Pr⁡(D=1,H=0|X)Pr⁡(D=0,H=0|X)=α+X′βX,     (1)
 MathType@MTEF@5@5@+=feaafiart1ev1aaatCvAUfKttLearuWrP9MDH5MBPbIqV92AaeXatLxBI9gBaebbnrfifHhDYfgasaacH8akY=wiFfYdH8Gipec8Eeeu0xXdbba9frFj0=OqFfea0dXdd9vqai=hGuQ8kuc9pgc9s8qqaq=dirpe0xb9q8qiLsFr0=vr0=vr0dc8meaabaqaciaacaGaaeqabaqabeGadaaakeaacyGGSbaBcqGGVbWBcqGGNbWzdaWcaaqaaiGbccfaqjabckhaYjabcIcaOiabdseaejabg2da9iabigdaXiabcYcaSiabdIeaijabg2da9iabicdaWiabcYha8jabdIfayjabcMcaPaqaaiGbccfaqjabckhaYjabcIcaOiabdseaejabg2da9iabicdaWiabcYcaSiabdIeaijabg2da9iabicdaWiabcYha8jabdIfayjabcMcaPaaacqGH9aqpiiGacqWFXoqycqGHRaWkcuWGybawgaqbaiab=j7aInaaBaaaleaacqWGybawaeqaaOGaeiilaWIaaCzcaiaaxMaadaqadiqaaiabigdaXaGaayjkaiaawMcaaaaa@593F@

log⁡Pr⁡(D=0,H=h|X)Pr⁡(D=0,H=0|X)=m(h,X;γ)     (2)
 MathType@MTEF@5@5@+=feaafiart1ev1aaatCvAUfKttLearuWrP9MDH5MBPbIqV92AaeXatLxBI9gBaebbnrfifHhDYfgasaacH8akY=wiFfYdH8Gipec8Eeeu0xXdbba9frFj0=OqFfea0dXdd9vqai=hGuQ8kuc9pgc9s8qqaq=dirpe0xb9q8qiLsFr0=vr0=vr0dc8meaabaqaciaacaGaaeqabaqabeGadaaakeaacyGGSbaBcqGGVbWBcqGGNbWzdaWcaaqaaiGbccfaqjabckhaYjabcIcaOiabdseaejabg2da9iabicdaWiabcYcaSiabdIeaijabg2da9iabdIgaOjabcYha8jabdIfayjabcMcaPaqaaiGbccfaqjabckhaYjabcIcaOiabdseaejabg2da9iabicdaWiabcYcaSiabdIeaijabg2da9iabicdaWiabcYha8jabdIfayjabcMcaPaaacqGH9aqpcqWGTbqBcqGGOaakcqWGObaAcqGGSaalcqWGybawcqGG7aWoiiGacqWFZoWzcqGGPaqkcaWLjaGaaCzcamaabmGabaGaeGOmaidacaGLOaGaayzkaaaaaa@5B25@

log⁡Pr⁡(D=1,H=h|X)Pr⁡(D=1,H=0|X)=βh+X′βhX+m(h,X;γ),h=0,...,M−1     (3)
 MathType@MTEF@5@5@+=feaafiart1ev1aaatCvAUfKttLearuWrP9MDH5MBPbIqV92AaeXatLxBI9gBaebbnrfifHhDYfgasaacH8akY=wiFfYdH8Gipec8Eeeu0xXdbba9frFj0=OqFfea0dXdd9vqai=hGuQ8kuc9pgc9s8qqaq=dirpe0xb9q8qiLsFr0=vr0=vr0dc8meaabaqaciaacaGaaeqabaqabeGadaaakeaafaqabeqacaaabaGagiiBaWMaei4Ba8Maei4zaC2aaSaaaeaacyGGqbaucqGGYbGCcqGGOaakcqWGebarcqGH9aqpcqaIXaqmcqGGSaalcqWGibascqGH9aqpcqWGObaAcqGG8baFcqWGybawcqGGPaqkaeaacyGGqbaucqGGYbGCcqGGOaakcqWGebarcqGH9aqpcqaIXaqmcqGGSaalcqWGibascqGH9aqpcqaIWaamcqGG8baFcqWGybawcqGGPaqkaaGaeyypa0dcciGae8NSdi2aaSbaaSqaaiabdIgaObqabaGccqGHRaWkcuWGybawgaqbaiab=j7aInaaBaaaleaacqWGObaAcqWGybawaeqaaOGaey4kaSIaemyBa0MaeiikaGIaemiAaGMaeiilaWIaemiwaGLaei4oaSJae83SdCMaeiykaKIaeiilaWcabaGaemiAaGMaeyypa0JaeGimaaJaeiilaWIaeiOla4IaeiOla4IaeiOla4IaeiilaWIaemyta0KaeyOeI0IaeGymaedaaiaaxMaacaWLjaWaaeWaceaacqaIZaWmaiaawIcacaGLPaaaaaa@7169@

with *β*_0 _= *β*_0*X *_= 0 and *m*(0, *X*; *γ*) = 0 for all *X*, where *m*(*H*, *X*; *γ*) is a known but arbitrary function of (*H*, *X*). Throughout the paper, a prime denotes matrix transposition.

To see the meanings of the parameters involved, rewrite (1)-(3) as

log⁡Pr⁡(D=1|X,H=0)Pr⁡(D=0|X,H=0)=α+X′βX     (4)
 MathType@MTEF@5@5@+=feaafiart1ev1aaatCvAUfKttLearuWrP9MDH5MBPbIqV92AaeXatLxBI9gBaebbnrfifHhDYfgasaacH8akY=wiFfYdH8Gipec8Eeeu0xXdbba9frFj0=OqFfea0dXdd9vqai=hGuQ8kuc9pgc9s8qqaq=dirpe0xb9q8qiLsFr0=vr0=vr0dc8meaabaqaciaacaGaaeqabaqabeGadaaakeaacyGGSbaBcqGGVbWBcqGGNbWzdaWcaaqaaiGbccfaqjabckhaYjabcIcaOiabdseaejabg2da9iabigdaXiabcYha8jabdIfayjabcYcaSiabdIeaijabg2da9iabicdaWiabcMcaPaqaaiGbccfaqjabckhaYjabcIcaOiabdseaejabg2da9iabicdaWiabcYha8jabdIfayjabcYcaSiabdIeaijabg2da9iabicdaWiabcMcaPaaacqGH9aqpiiGacqWFXoqycqGHRaWkcuWGybawgaqbaiab=j7aInaaBaaaleaacqWGybawaeqaaOGaaCzcaiaaxMaadaqadiqaaiabisda0aGaayjkaiaawMcaaaaa@5865@

log⁡Pr⁡(H=h|X,D=0)Pr⁡(H=0|X,D=0)=m(h,X;γ),h=0,...,M−1     (5)
 MathType@MTEF@5@5@+=feaafiart1ev1aaatCvAUfKttLearuWrP9MDH5MBPbIqV92AaeXatLxBI9gBaebbnrfifHhDYfgasaacH8akY=wiFfYdH8Gipec8Eeeu0xXdbba9frFj0=OqFfea0dXdd9vqai=hGuQ8kuc9pgc9s8qqaq=dirpe0xb9q8qiLsFr0=vr0=vr0dc8meaabaqaciaacaGaaeqabaqabeGadaaakeaafaqabeqacaaabaGagiiBaWMaei4Ba8Maei4zaC2aaSaaaeaacyGGqbaucqGGYbGCcqGGOaakcqWGibascqGH9aqpcqWGObaAcqGG8baFcqWGybawcqGGSaalcqWGebarcqGH9aqpcqaIWaamcqGGPaqkaeaacyGGqbaucqGGYbGCcqGGOaakcqWGibascqGH9aqpcqaIWaamcqGG8baFcqWGybawcqGGSaalcqWGebarcqGH9aqpcqaIWaamcqGGPaqkaaGaeyypa0JaemyBa0MaeiikaGIaemiAaGMaeiilaWIaemiwaGLaei4oaSdcciGae83SdCMaeiykaKIaeiilaWcabaGaemiAaGMaeyypa0JaeGimaaJaeiilaWIaeiOla4IaeiOla4IaeiOla4IaeiilaWIaemyta0KaeyOeI0IaeGymaedaaiaaxMaacaWLjaWaaeWaceaacqaI1aqnaiaawIcacaGLPaaaaaa@66D1@

log⁡Pr⁡(H=h|X,D=1)Pr⁡(H=0|X,D=1)=βh+X′βhX+m(h,X;γ),h=0,...,M−1,     (6)
 MathType@MTEF@5@5@+=feaafiart1ev1aaatCvAUfKttLearuWrP9MDH5MBPbIqV92AaeXatLxBI9gBaebbnrfifHhDYfgasaacH8akY=wiFfYdH8Gipec8Eeeu0xXdbba9frFj0=OqFfea0dXdd9vqai=hGuQ8kuc9pgc9s8qqaq=dirpe0xb9q8qiLsFr0=vr0=vr0dc8meaabaqaciaacaGaaeqabaqabeGadaaakeaafaqabeqacaaabaGagiiBaWMaei4Ba8Maei4zaC2aaSaaaeaacyGGqbaucqGGYbGCcqGGOaakcqWGibascqGH9aqpcqWGObaAcqGG8baFcqWGybawcqGGSaalcqWGebarcqGH9aqpcqaIXaqmcqGGPaqkaeaacyGGqbaucqGGYbGCcqGGOaakcqWGibascqGH9aqpcqaIWaamcqGG8baFcqWGybawcqGGSaalcqWGebarcqGH9aqpcqaIXaqmcqGGPaqkaaGaeyypa0dcciGae8NSdi2aaSbaaSqaaiabdIgaObqabaGccqGHRaWkcuWGybawgaqbaiab=j7aInaaBaaaleaacqWGObaAcqWGybawaeqaaOGaey4kaSIaemyBa0MaeiikaGIaemiAaGMaeiilaWIaemiwaGLaei4oaSJae83SdCMaeiykaKIaeiilaWcabaGaemiAaGMaeyypa0JaeGimaaJaeiilaWIaeiOla4IaeiOla4IaeiOla4IaeiilaWIaemyta0KaeyOeI0IaeGymaedaaiabcYcaSiaaxMaacaWLjaWaaeWaceaacqaI2aGnaiaawIcacaGLPaaaaaa@724F@

*β*_0 _= *β*_0*X *_= 0 and *m*(0, *X*; *γ*) = 0 for all *X*. We can see that the parameters *α *and *β*_*X *_quantify respectively the baseline disease risk and the effect of environmental exposures on the disease risk for subjects with the reference haplotype pair. The function *m*(*H*, *X*; *γ*) specifies the distribution of the haplotype pairs among the control population given the covariates *X*. The parameters *β*_*h *_and *β*_*hX *_(*h *= 1,..., *M *- 1) measure, in the retrospective odds-ratio scale, respectively the main effect of the haplotype pair *h *(relative to the reference pair) and the interaction between the haplotype pair *h *and the covariates *X*. Note that although *β*_*h *_and *β*_*hX *_are specified through the retrospective model Pr(*H*|*X*, *D*), they are equivalent to the corresponding parameters specified in the prospective disease model Pr(*D*|*X*, *H*). In fact, according to (1)-(3), the joint probability of (*D*, *H*) given *X *is obtained as

Pr⁡(D=i,H=h|X,θ)=exp⁡{Λih(X;θ}∑i=01∑h=0M−1exp⁡{Λih(X;θ)},     (7)
 MathType@MTEF@5@5@+=feaafiart1ev1aaatCvAUfKttLearuWrP9MDH5MBPbIqV92AaeXatLxBI9gBaebbnrfifHhDYfgasaacH8akY=wiFfYdH8Gipec8Eeeu0xXdbba9frFj0=OqFfea0dXdd9vqai=hGuQ8kuc9pgc9s8qqaq=dirpe0xb9q8qiLsFr0=vr0=vr0dc8meaabaqaciaacaGaaeqabaqabeGadaaakeaacyGGqbaucqGGYbGCcqGGOaakcqWGebarcqGH9aqpcqWGPbqAcqGGSaalcqWGibascqGH9aqpcqWGObaAcqGG8baFcqWGybawcqGGSaaliiGacqWF4oqCcqGGPaqkcqGH9aqpdaWcaaqaaiGbcwgaLjabcIha4jabcchaWnaacmqabaGaeu4MdW0aaSbaaSqaaiabdMgaPjabdIgaObqabaGccqGGOaakcqWGybawcqGG7aWocqWF4oqCaiaawUhacaGL9baaaeaadaaeWaqaamaaqadabaGagiyzauMaeiiEaGNaeiiCaa3aaiWabeaacqqHBoatdaWgaaWcbaGaemyAaKMaemiAaGgabeaakiabcIcaOiabdIfayjabcUda7iab=H7aXjabcMcaPaGaay5Eaiaaw2haaaWcbaGaemiAaGMaeyypa0JaeGimaadabaGaemyta0KaeyOeI0IaeGymaedaniabggHiLdaaleaacqWGPbqAcqGH9aqpcqaIWaamaeaacqaIXaqma0GaeyyeIuoaaaGccqGGSaalcaWLjaGaaCzcamaabmGabaGaeG4naCdacaGLOaGaayzkaaaaaa@7253@

where

Λ_*ih *_(*X*; *θ*) = *i *(*α *+ *β*_*h *_+ *X*' *β*_*X *_+ *X*' *β*_*hX*_) + *m*(*h*, *X*; *γ*),

*θ *= (*α*, *β*_*X*_, *β*_*h*_, *β*_*hX*_, *γ*; *h *= 0,..., *M *- 1) with *β*_0 _= *β*_0*X *_= 0 and *m*(0, *X*; *γ*) = 0 for all *X*. The prospective disease model Pr(*D*|*H*, *X*) is then given by

log⁡Pr⁡(D=1|X,H=h)Pr⁡(D=0|X,H=h)=α+βh+X′βX+X′βhX.     (8)
 MathType@MTEF@5@5@+=feaafiart1ev1aaatCvAUfKttLearuWrP9MDH5MBPbIqV92AaeXatLxBI9gBaebbnrfifHhDYfgasaacH8akY=wiFfYdH8Gipec8Eeeu0xXdbba9frFj0=OqFfea0dXdd9vqai=hGuQ8kuc9pgc9s8qqaq=dirpe0xb9q8qiLsFr0=vr0=vr0dc8meaabaqaciaacaGaaeqabaqabeGadaaakeaacyGGSbaBcqGGVbWBcqGGNbWzdaWcaaqaaiGbccfaqjabckhaYjabcIcaOiabdseaejabg2da9iabigdaXiabcYha8jabdIfayjabcYcaSiabdIeaijabg2da9iabdIgaOjabcMcaPaqaaiGbccfaqjabckhaYjabcIcaOiabdseaejabg2da9iabicdaWiabcYha8jabdIfayjabcYcaSiabdIeaijabg2da9iabdIgaOjabcMcaPaaacqGH9aqpiiGacqWFXoqycqGHRaWkcqWFYoGydaWgaaWcbaGaemiAaGgabeaakiabgUcaRiqbdIfayzaafaGae8NSdi2aaSbaaSqaaiabdIfaybqabaGccqGHRaWkcuWGybawgaqbaiab=j7aInaaBaaaleaacqWGObaAcqWGybawaeqaaOGaeiOla4IaaCzcaiaaxMaadaqadiqaaiabiIda4aGaayjkaiaawMcaaaaa@64BF@

Therefore, all the association (odds ratio) parameters regarding the associations between haplotype-environment factors and disease phenotype in the proposed model are equivalent to those specified in a prospective disease risk model. Note that the models (4)–(6) are equivalent to the prospective disease risk model (8) together with the model (5) for the haplotype-pair distribution in the controls.

The proposed modeling setup can allow flexible modeling of the effects of haplotype pairs. Take *β*_*h *_= Z′h
 MathType@MTEF@5@5@+=feaafiart1ev1aaatCvAUfKttLearuWrP9MDH5MBPbIqV92AaeXatLxBI9gBaebbnrfifHhDYfgasaacH8akY=wiFfYdH8Gipec8Eeeu0xXdbba9frFj0=OqFfea0dXdd9vqai=hGuQ8kuc9pgc9s8qqaq=dirpe0xb9q8qiLsFr0=vr0=vr0dc8meaabaqaciaacaGaaeqabaqabeGadaaakeaacuWGAbGwgaqbamaaBaaaleaacqWGObaAaeqaaaaa@2F7A@*β*_*hap *_where *Z*_*h *_is a vector of coded values for the haplotype pair *h *according to a certain genetic law, and *β*_*hap *_is the vector of associated regression coefficients quantifying the marginal haplotype effects relative to the reference haplotype. For example, to evaluate the effect of a specific haplotype ħ_*_, we can set *Z*_*h *_= *I *(*h*^1 ^= ħ _*_) *I *(*h*^2 ^= ħ_*_) for a recessive genetic law, *Z*_*h *_= *I *(*h*^1 ^= ħ_*_)+ *I *(*h*^2 ^= ħ_*_) - *I *(*h*^*l *^= ħ_*_) *I *(*h*^2 ^= ħ_*_) for a dominant law, and *Z*_*h *_= *I *(*h*^*l *^= ħ_*_) + *I *(*h*^2 ^= ħ_*_) for a multiplicative law, where *I *(*A*) is the indicator function which equals one if the event *A *occurs and equals zero otherwise. More examples for the specification of the genetic effects can be found in Epstein and Satten [7] and Zhao et al. [12]. Similarly, by taking βhX*
 MathType@MTEF@5@5@+=feaafiart1ev1aaatCvAUfKttLearuWrP9MDH5MBPbIqV92AaeXatLxBI9gBaebbnrfifHhDYfgasaacH8akY=wiFfYdH8Gipec8Eeeu0xXdbba9frFj0=OqFfea0dXdd9vqai=hGuQ8kuc9pgc9s8qqaq=dirpe0xb9q8qiLsFr0=vr0=vr0dc8meaabaqaciaacaGaaeqabaqabeGadaaakeaaiiGacqWFYoGydaWgaaWcbaGaemiAaGMaemiwaG1aaSbaaWqaaiabcQcaQaqabaaaleqaaaaa@3226@ = X_*_Z′h
 MathType@MTEF@5@5@+=feaafiart1ev1aaatCvAUfKttLearuWrP9MDH5MBPbIqV92AaeXatLxBI9gBaebbnrfifHhDYfgasaacH8akY=wiFfYdH8Gipec8Eeeu0xXdbba9frFj0=OqFfea0dXdd9vqai=hGuQ8kuc9pgc9s8qqaq=dirpe0xb9q8qiLsFr0=vr0=vr0dc8meaabaqaciaacaGaaeqabaqabeGadaaakeaacuWGAbGwgaqbamaaBaaaleaacqWGObaAaeqaaaaa@2F7A@*β*_*int *_we can evaluate the interactions *β*_*int *_between haplotypes and a chosen environmental covariate X_*_. Although the above examples focus on effects associated with one single haplotype, using a collection of *Z*_*h *_variables for multiple causal haplotypes we can fit extensive models with multiple causal haplotypes; see Results, Application to the hypertriglyceridemia study for an illustration.

Various models for the haplotype-pair distribution in the control population can also be incorporated into the proposed model with suitable specifications of the function *m*(*H*, *X*; *γ*). Note that a saturated model for the haplotype-pair distribution is not identifiable from the unphased genotype data [7], so certain modeling constraints must be imposed to ensure identifiability. One convenient specification is to assume that, in the control population, the haplotype-pair distribution is independent of the covariates *X *and satisfies the Hardy Weinberg equilibrium (HWE), namely.

Pr(*H*^1 ^= *h*_*j*_, *H*^2 ^= *ħ*_*k*_|*X*, *D *= 0) = Pr(*H*^1 ^= *h*_*j*_, *H*^2 ^= *ħ*_*k*_|*D *= 0) = *π*_*j*_*π*_*k*_, *j*, *k *= 0, ..., *J *- 1,

where *π*_*j *_is the marginal frequency of haplotype *ħ*_*j *_in the control population. Such a distribution corresponds to the specification *m*(*H*, *X*; *γ*) = W′H
 MathType@MTEF@5@5@+=feaafiart1ev1aaatCvAUfKttLearuWrP9MDH5MBPbIqV92AaeXatLxBI9gBaebbnrfifHhDYfgasaacH8akY=wiFfYdH8Gipec8Eeeu0xXdbba9frFj0=OqFfea0dXdd9vqai=hGuQ8kuc9pgc9s8qqaq=dirpe0xb9q8qiLsFr0=vr0=vr0dc8meaabaqaciaacaGaaeqabaqabeGadaaakeaacuWGxbWvgaqbamaaBaaaleaacqWGibasaeqaaaaa@2F34@*γ*, where *W*_*H *_is a *J*-vector with the *j*th component given by *I *(*H*^1^= *ħ*_*j*_) + *I *(*H*^2 ^= *ħ*_*j*_), and the *j*th component of *γ *is related to *π *by *γ*_*j *_= log (*π*_*j*_/*π*_0_), *j *= 0,..., *J *- 1. A more general assumption is to allow the HWE holds only within each of the strata defined by some categorical covariate *S*, and the corresponding specification of *m*(*H*, *X*; *γ*) can be expressed as *m*(*H*, *X*; *γ*) = *m*(*H*, *S*; *γ*) = W′H
 MathType@MTEF@5@5@+=feaafiart1ev1aaatCvAUfKttLearuWrP9MDH5MBPbIqV92AaeXatLxBI9gBaebbnrfifHhDYfgasaacH8akY=wiFfYdH8Gipec8Eeeu0xXdbba9frFj0=OqFfea0dXdd9vqai=hGuQ8kuc9pgc9s8qqaq=dirpe0xb9q8qiLsFr0=vr0=vr0dc8meaabaqaciaacaGaaeqabaqabeGadaaakeaacuWGxbWvgaqbamaaBaaaleaacqWGibasaeqaaaaa@2F34@*γ*_*S*_, where *W*_*H *_is denned as above and *γ*_*S *_is a stratum-specific parameter with the strata determined by *S*. For example, when population stratification exists, then the strata *S *can be defined by the subpopulations or ethnicity groups to account for the violation of HWE caused by population stratification. Another way to relax the HWE assumption is to introduce the fixation parameter [13] into the model *m*(*H*, *X*) for Pr(*H*|*X*, *D *= 0); see Satten and Epstein [14] for details.

### Maximum likelihood estimation

Let *N*_1 _and *N*_0 _be respectively the number of cases and controls in the sample, and *N *= *N*_1 _+ *N*_0 _the total sample size. For the *u*th subject in the case-control sample, *u *= 1,..., *N*, suppose that the observed data are (*D*_*u*_, *G*_*u*_, *X*_*u*_), including the disease status *D*_*u*_, the unphased genotype *G*_*u*_, and the environmental covariates *X*_*u*_. The haplotype data *H*_*u *_cannot be uniquely determined from the unphased genotype data *G*_*u *_if the *u*th subject is heterozygous at more than one SNP.

Let *S*(*G*) be the set of labels of haplotype pairs that are consistent with unphased genotype *G*. Using (7), the probability of (*D*, *G*) given *X *can be expressed as

Pr⁡(D,G|X;θ)=∑h∈S(G)Pr⁡(D,H=h|X;θ)=exp⁡{D(α+βX)}∑h∈S(G)exp⁡{D(βh+βhX)+m(H=h,X;γ)}∑d=01∑h=0M−1exp⁡{d(α+βX+βh+βhX)+m(H=h,X;γ}.     (9)
 MathType@MTEF@5@5@+=feaafiart1ev1aaatCvAUfKttLearuWrP9MDH5MBPbIqV92AaeXatLxBI9gBamrtHrhAL1wy0L2yHvtyaeHbnfgDOvwBHrxAJfwnaebbnrfifHhDYfgasaacH8akY=wiFfYdH8Gipec8Eeeu0xXdbba9frFj0=OqFfea0dXdd9vqai=hGuQ8kuc9pgc9s8qqaq=dirpe0xb9q8qiLsFr0=vr0=vr0dc8meaabaqaciaacaGaaeqabaWaaeGaeaaakeaafaqadeGabaaabaGagiiuaaLaeiOCaiNaeiikaGIaemiraqKaeiilaWIaem4raCKaeiiFaWNaemiwaGLaei4oaSdcciGae8hUdeNaeiykaKIaeyypa0ZaaabuaeaacyGGqbaucqGGYbGCcqGGOaakcqWGebarcqGGSaalcqWGibascqGH9aqpcqWGObaAcqGG8baFcqWGybawcqGG7aWocqWF4oqCcqGGPaqkaSqaaiabdIgaOjabgIGioJWaaiab+jr8tjabcIcaOiabdEeahjabcMcaPaqab0GaeyyeIuoaaOqaaiabg2da9maalaaabaGagiyzauMaeiiEaGNaeiiCaa3aaiWaaeaacqWGebarcqGGOaakcqWFXoqycqGHRaWkcqWFYoGydaWgaaWcbaGaemiwaGfabeaakiabcMcaPaGaay5Eaiaaw2haamaaqababaGagiyzauMaeiiEaGNaeiiCaa3aaiWaaeaacqWGebarcqGGOaakcqWFYoGydaWgaaWcbaGaemiAaGgabeaakiabgUcaRiab=j7aInaaBaaaleaacqWGObaAcqWGybawaeqaaOGaeiykaKIaey4kaSIaemyBa0MaeiikaGIaemisaGKaeyypa0JaemiAaGMaeiilaWIaemiwaGLaei4oaSJae83SdCMaeiykaKcacaGL7bGaayzFaaaaleaacqWGObaAcqGHiiIZcqGFse=ucqGGOaakcqWGhbWrcqGGPaqkaeqaniabggHiLdaakeaadaaeWaqaamaaqadabaGagiyzauMaeiiEaGNaeiiCaahaleaacqWGObaAcqGH9aqpcqaIWaamaeaacqWGnbqtcqGHsislcqaIXaqma0GaeyyeIuoakmaacmaabaGaemizaqMaeiikaGIae8xSdeMaey4kaSIae8NSdi2aaSbaaSqaaiabdIfaybqabaGccqGHRaWkcqWFYoGydaWgaaWcbaGaemiAaGgabeaakiabgUcaRiab=j7aInaaBaaaleaacqWGObaAcqWGybawaeqaaOGaeiykaKIaey4kaSIaemyBa0MaeiikaGIaemisaGKaeyypa0JaemiAaGMaeiilaWIaemiwaGLaei4oaSJae83SdCgacaGL7bGaayzFaaaaleaacqWGKbazcqGH9aqpcqaIWaamaeaacqaIXaqma0GaeyyeIuoaaaGccqGGUaGlaaGaaCzcaiaaxMaadaqadaqaaiabiMda5aGaayjkaiaawMcaaaaa@CAD7@

In the following discussions we will assume that models for *β*_*H*_, *β*_*HX *_and *m*(*H*, *X*; *γ*) are specified in such a way that all the parameters involved are identifiable from prospective studies. In Appendix 1 we provide identifiability conditions of *β*_*H *_and *β*_*HX *_for a given model *m*(*H*, *X*; *γ*).

The loglikelihood for the observed data (Du,Gu,Xu)u=1N
 MathType@MTEF@5@5@+=feaafiart1ev1aaatCvAUfKttLearuWrP9MDH5MBPbIqV92AaeXatLxBI9gBaebbnrfifHhDYfgasaacH8akY=wiFfYdH8Gipec8Eeeu0xXdbba9frFj0=OqFfea0dXdd9vqai=hGuQ8kuc9pgc9s8qqaq=dirpe0xb9q8qiLsFr0=vr0=vr0dc8meaabaqaciaacaGaaeqabaqabeGadaaakeaacqGGOaakcqWGebardaWgaaWcbaGaemyDauhabeaakiabcYcaSiabdEeahnaaBaaaleaacqWG1bqDaeqaaOGaeiilaWIaemiwaG1aaSbaaSqaaiabdwha1bqabaGccqGGPaqkdaqhaaWcbaGaemyDauNaeyypa0JaeGymaedabaGaemOta4eaaaaa@3D35@ in a case-control sample is given by

∑u=1Nlog⁡Pr⁡(Gu,Xu|Du;θ)=∑u=1Nlog⁡{Pr⁡(Du,Gu|Xu;θ)p(Xu)/Pr⁡(Du)}=∑u=1Nlog⁡{∑h∈S(Gu)Pr⁡(Du,H=h|Xu;θ)p(Xu)/Pr⁡(Du)}     (10)
 MathType@MTEF@5@5@+=feaafiart1ev1aaatCvAUfKttLearuWrP9MDH5MBPbIqV92AaeXatLxBI9gBamrtHrhAL1wy0L2yHvtyaeHbnfgDOvwBHrxAJfwnaebbnrfifHhDYfgasaacH8akY=wiFfYdH8Gipec8Eeeu0xXdbba9frFj0=OqFfea0dXdd9vqai=hGuQ8kuc9pgc9s8qqaq=dirpe0xb9q8qiLsFr0=vr0=vr0dc8meaabaqaciaacaGaaeqabaWaaeGaeaaakeaafaqaaeWacaaabaaabaWaaabCaeaacyGGSbaBcqGGVbWBcqGGNbWzaSqaaiabdwha1jabg2da9iabigdaXaqaaiabd6eaobqdcqGHris5aOGagiiuaaLaeiOCaiNaeiikaGIaem4raC0aaSbaaSqaaiabdwha1bqabaGccqGGSaalcqWGybawdaWgaaWcbaGaemyDauhabeaakiabcYha8jabdseaenaaBaaaleaacqWG1bqDaeqaaOGaei4oaSdcciGae8hUdeNaeiykaKcabaGaeyypa0dabaWaaabCaeaacyGGSbaBcqGGVbWBcqGGNbWzdaGadaqaaiGbccfaqjabckhaYjabcIcaOiabdseaenaaBaaaleaacqWG1bqDaeqaaOGaeiilaWIaem4raC0aaSbaaSqaaiabdwha1bqabaGccqGG8baFcqWGybawdaWgaaWcbaGaemyDauhabeaakiabcUda7iab=H7aXjabcMcaPiabdchaWjabcIcaOiabdIfaynaaBaaaleaacqWG1bqDaeqaaOGaeiykaKIaei4la8IagiiuaaLaeiOCaiNaeiikaGIaemiraq0aaSbaaSqaaiabdwha1bqabaGccqGGPaqkaiaawUhacaGL9baaaSqaaiabdwha1jabg2da9iabigdaXaqaaiabd6eaobqdcqGHris5aaGcbaGaeyypa0dabaWaaabCaeaacyGGSbaBcqGGVbWBcqGGNbWzdaGadaqaamaaqafabaGagiiuaaLaeiOCaiNaeiikaGIaemiraq0aaSbaaSqaaiabdwha1bqabaGccqGGSaalcqWGibascqGH9aqpcqWGObaAcqGG8baFcqWGybawdaWgaaWcbaGaemyDauhabeaakiabcUda7iab=H7aXjabcMcaPiabdchaWjabcIcaOiabdIfaynaaBaaaleaacqWG1bqDaeqaaOGaeiykaKIaei4la8IagiiuaaLaeiOCaiNaeiikaGIaemiraq0aaSbaaSqaaiabdwha1bqabaGccqGGPaqkaSqaaiabdIgaOjabgIGioJWaaiab+jr8tjabcIcaOiabdEeahnaaBaaameaacqWG1bqDaeqaaSGaeiykaKcabeqdcqGHris5aaGccaGL7bGaayzFaaaaleaacqWG1bqDcqGH9aqpcqaIXaqmaeaacqWGobGta0GaeyyeIuoaaaGccaWLjaGaaCzcamaabmaabaGaeGymaeJaeGimaadacaGLOaGaayzkaaaaaa@BF7C@

where Pr(*D*, *H*|*X*; *θ*) is given by (7), *p*(*X*) denotes the marginal density function of *X*, and Pr⁡(D)=∫x∑h=0M−1Pr⁡(D,H=h|X=x)p(x)dx
 MathType@MTEF@5@5@+=feaafiart1ev1aaatCvAUfKttLearuWrP9MDH5MBPbIqV92AaeXatLxBI9gBaebbnrfifHhDYfgasaacH8akY=wiFfYdH8Gipec8Eeeu0xXdbba9frFj0=OqFfea0dXdd9vqai=hGuQ8kuc9pgc9s8qqaq=dirpe0xb9q8qiLsFr0=vr0=vr0dc8meaabaqaciaacaGaaeqabaqabeGadaaakeaacyGGqbaucqGGYbGCcqGGOaakcqWGebarcqGGPaqkcqGH9aqpdaWdraqaamaaqadabaGagiiuaaLaeiOCaiNaeiikaGIaemiraqKaeiilaWIaemisaGKaeyypa0JaemiAaGMaeiiFaWNaemiwaGLaeyypa0JaemiEaGNaeiykaKIaemiCaaNaeiikaGIaemiEaGNaeiykaKIaemizaqMaemiEaGhaleaacqWGObaAcqGH9aqpcqaIWaamaeaacqWGnbqtcqGHsislcqaIXaqma0GaeyyeIuoaaSqaaiabdIha4bqab0Gaey4kIipaaaa@5524@ We leave *p*(*X*) fully unspecified. Using the profile likelihood approach to profile out the nuisance parameter *p*(*X*), the retrospective loglikelihood (10) can be translated into a prospective loglikelihood.

### The profile likelihood

Let α^* ^= *α *+ log(*ν*_*1*_/*ν*_0_) with *ν*_*i *_= *N*_*i*_/{*N*Pr(*D *= *i*)}, *i *= 0,1. Define θ^* ^as *θ *with *α *replaced by α^*^. Under the multinomial logistic model Pr(*D*, *H*|*X*; *θ*) given in (7), the retrospective loglikelihood for the unphased genotype data in a case-control sample can be shown to be equivalent to the prospective loglikelihood

LP=∑u=1Nlog⁡Pr⁡∗(Du,Gu|Xu;θ∗)=∑u=1Nlog⁡{∑h∈S(Gu)Pr⁡∗(Du,H=h|Xu;θ∗)},
 MathType@MTEF@5@5@+=feaafiart1ev1aaatCvAUfKttLearuWrP9MDH5MBPbIqV92AaeXatLxBI9gBamrtHrhAL1wy0L2yHvtyaeHbnfgDOvwBHrxAJfwnaebbnrfifHhDYfgasaacH8akY=wiFfYdH8Gipec8Eeeu0xXdbba9frFj0=OqFfea0dXdd9vqai=hGuQ8kuc9pgc9s8qqaq=dirpe0xb9q8qiLsFr0=vr0=vr0dc8meaabaqaciaacaGaaeqabaWaaeGaeaaakeaacqWGmbatdaWgaaWcbaGaemiuaafabeaakiabg2da9maaqahabaGagiiBaWMaei4Ba8Maei4zaCMagiiuaaLaeiOCai3aaWbaaSqabeaacqGHxiIkaaGccqGGOaakcqWGebardaWgaaWcbaGaemyDauhabeaakiabcYcaSiabdEeahnaaBaaaleaacqWG1bqDaeqaaOGaeiiFaWNaemiwaG1aaSbaaSqaaiabdwha1bqabaGccqGG7aWoiiGacqWF4oqCdaahaaWcbeqaaiabgEHiQaaakiabcMcaPaWcbaGaemyDauNaeyypa0JaeGymaedabaGaemOta4eaniabggHiLdGccqGH9aqpdaaeWbqaaiGbcYgaSjabc+gaVjabcEgaNnaacmaabaWaaabuaeaacyGGqbaucqGGYbGCdaahaaWcbeqaaiabgEHiQaaakiabcIcaOiabdseaenaaBaaaleaacqWG1bqDaeqaaOGaeiilaWIaemisaGKaeyypa0JaemiAaGMaeiiFaWNaemiwaG1aaSbaaSqaaiabdwha1bqabaGccqGG7aWocqWF4oqCdaahaaWcbeqaaiabgEHiQaaakiabcMcaPaWcbaGaemiAaGMaeyicI4mcdaGae4NeXpLaeiikaGIaem4raC0aaSbaaWqaaiabdwha1bqabaWccqGGPaqkaeqaniabggHiLdaakiaawUhacaGL9baacqGGSaalaSqaaiabdwha1jabg2da9iabigdaXaqaaiabd6eaobqdcqGHris5aaaa@8807@

where Pr^*^(*D*, *H*|*X*; θ^*^) is defined as Pr(*D*, *H*|*X*; *θ*) with *α *replaced by α^*^. The derivation is relegated to Appendix 2.

This result is parallel to the classic one given by Prentice and Pyke [2] when haplotype data are directly observed. Note that in the prospective likelihood *L*_*P *_the original intercept *α *is absorbed into α^* ^and does not appear as a separate parameter. Hence *α *is not identifiable and estimable, unless supplementary information on the population disease prevalence Pr(*D *= 1) is utilized to separate *α *from α^*^. Further, following the derivation in Prentice and Pyke [2] or Scott and Wild [15], we can show that the estimated covariance matrix for all the parameters except α^* ^can be obtained from the corresponding submatrix of the observed information matrix based on *L*_*P*_. Therefore, under the proposed modeling setup the maximum likelihood inference on all the parameters except *α *can be based on the prospective loglikelihood *L*_*P*_, with the case-control sampling design being ignored. Another consequence is that, using the result of Scott and Wild [15], using external information on Pr(*D *= 1) in our proposal only affects the inference of the intercept parameter and does not affect the efficiency of estimates for all the other parameters.

### EM algorithm

The maximum likelihood estimation based on *L*_*P *_can be simply implemented by the EM algorithm. Write *∂L*_*P*_/∂θ^* ^= ∑_*u *_Ψ_*u *_(θ^*^), and let

ΨuC(θ∗)=∂∂θ∗log⁡Pr⁡∗(Du,Hu|Xu;θ∗),
 MathType@MTEF@5@5@+=feaafiart1ev1aaatCvAUfKttLearuWrP9MDH5MBPbIqV92AaeXatLxBI9gBaebbnrfifHhDYfgasaacH8akY=wiFfYdH8Gipec8Eeeu0xXdbba9frFj0=OqFfea0dXdd9vqai=hGuQ8kuc9pgc9s8qqaq=dirpe0xb9q8qiLsFr0=vr0=vr0dc8meaabaqaciaacaGaaeqabaqabeGadaaakeaacqqHOoqwdaqhaaWcbaGaemyDauhabaGaem4qameaaOGaeiikaGccciGae8hUde3aaWbaaSqabeaacqGHxiIkaaGccqGGPaqkcqGH9aqpdaWcaaqaaiabgkGi2cqaaiabgkGi2kab=H7aXnaaCaaaleqabaGaey4fIOcaaaaakiGbcYgaSjabc+gaVjabcEgaNjGbccfaqjabckhaYnaaCaaaleqabaGaey4fIOcaaOGaeiikaGIaemiraq0aaSbaaSqaaiabdwha1bqabaGccqGGSaalcqWGibasdaWgaaWcbaGaemyDauhabeaakiabcYha8jabdIfaynaaBaaaleaacqWG1bqDaeqaaOGaei4oaSJae8hUde3aaWbaaSqabeaacqGHxiIkaaGccqGGPaqkcqGGSaalaaa@5541@

which is the *u*th subject's "complete-data" score function based on Pr^*^(*D*, *H*|*X*; θ^*^); see Appendix 3 for its explicit expression. It can be seen that

Ψu(θ∗)=∑h∈S(Gu)∂Pr⁡∗(Du,Hu=h|Xu;θ∗)/∂θ∗∑h∈S(Gu)Pr⁡∗(Du,Hu=h|Xu;θ∗)=∑h∈S(Gu)ΨuC(θ)Pr⁡∗(Du,Hu=h|Xu;θ∗)∑h∈S(Gu)Pr⁡∗(Du,Hu=h|Xu;θ∗)=E{ΨuC(θ∗)|Du,Gu,Xu;θ∗},     (11)
 MathType@MTEF@5@5@+=feaafiart1ev1aaatCvAUfKttLearuWrP9MDH5MBPbIqV92AaeXatLxBI9gBamrtHrhAL1wy0L2yHvtyaeHbnfgDOvwBHrxAJfwnaebbnrfifHhDYfgasaacH8akY=wiFfYdH8Gipec8Eeeu0xXdbba9frFj0=OqFfea0dXdd9vqai=hGuQ8kuc9pgc9s8qqaq=dirpe0xb9q8qiLsFr0=vr0=vr0dc8meaabaqaciaacaGaaeqabaWaaeGaeaaakeGacmaZgmachuaabeqadmaaaeaacqqHOoqwdaWgaaWcbaGaemyDauhabeaakiabcIcaOGGaciab=H7aXnaaCaaaleqabaGaey4fIOcaaOGaeiykaKcabaGaeyypa0dabaWaaSaaaeaadaaeqaqaaiabgkGi2kGbccfaqjabckhaYnaaCaaaleqabaGaey4fIOcaaaqaaiabdIgaOjabgIGioJWaaiab+jr8tjabcIcaOiabdEeahnaaBaaameaacqWG1bqDaeqaaSGaeiykaKcabeqdcqGHris5aOGaeiikaGIaemiraq0aaSbaaSqaaiabdwha1bqabaGccqGGSaalcqWGibasdaWgaaWcbaGaemyDauhabeaakiabg2da9iabdIgaOjabcYha8jabdIfaynaaBaaaleaacqWG1bqDaeqaaOGaei4oaSJae8hUde3aaWbaaSqabeaacqGHxiIkaaGccqGGPaqkcqGGVaWlcqGHciITcqWF4oqCdaahaaWcbeqaaiabgEHiQaaaaOqaamaaqababaGagiiuaaLaeiOCai3aaWbaaSqabeaacqGHxiIkaaaabaGaemiAaGMaeyicI4Sae4NeXpLaeiikaGIaem4raC0aaSbaaWqaaiabdwha1bqabaWccqGGPaqkaeqaniabggHiLdGccqGGOaakcqWGebardaWgaaWcbaGaemyDauhabeaakiabcYcaSiabdIeainaaBaaaleaacqWG1bqDaeqaaOGaeyypa0JaemiAaGMaeiiFaWNaemiwaG1aaSbaaSqaaiabdwha1bqabaGccqGG7aWocqWF4oqCdaahaaWcbeqaaiabgEHiQaaakiabcMcaPaaaaeaaaeaacqGH9aqpaeaadaWcaaqaamaaqababaGaeuiQdK1aa0baaSqaaiabdwha1bqaaiabdoeadbaakiabcIcaOiab=H7aXjabcMcaPiGbccfaqjabckhaYnaaCaaaleqabaGaey4fIOcaaaqaaiabdIgaOjabgIGiolab+jr8tjabcIcaOiabdEeahnaaBaaameaacqWG1bqDaeqaaSGaeiykaKcabeqdcqGHris5aOGaeiikaGIaemiraq0aaSbaaSqaaiabdwha1bqabaGccqGGSaalcqWGibasdaWgaaWcbaGaemyDauhabeaakiabg2da9iabdIgaOjabcYha8jabdIfaynaaBaaaleaacqWG1bqDaeqaaOGaei4oaSJae8hUde3aaWbaaSqabeaacqGHxiIkaaGccqGGPaqkaeaadaaeqaqaaiGbccfaqjabckhaYnaaCaaaleqabaGaey4fIOcaaaqaaiabdIgaOjabgIGiolab+jr8tjabcIcaOiabdEeahnaaBaaameaacqWG1bqDaeqaaSGaeiykaKcabeqdcqGHris5aOGaeiikaGIaemiraq0aaSbaaSqaaiabdwha1bqabaGccqGGSaalcqWGibasdaWgaaWcbaGaemyDauhabeaakiabg2da9iabdIgaOjabcYha8jabdIfaynaaBaaaleaacqWG1bqDaeqaaOGaei4oaSJae8hUde3aaWbaaSqabeaacqGHxiIkaaGccqGGPaqkaaaabaaabaGaeyypa0dabiqae0o=cqWGfbqrdaGadaqaaiabfI6aznaaDaaaleaacqWG1bqDaeaacqWGdbWqaaGccqGGOaakcqWF4oqCdaahaaWcbeqaaiabgEHiQaaakiabcMcaPiabcYha8jabdseaenaaBaaaleaacqWG1bqDaeqaaOGaeiilaWIaem4raC0aaSbaaSqaaiabdwha1bqabaGccqGGSaalcqWGybawdaWgaaWcbaGaemyDauhabeaakiabcUda7iab=H7aXnaaCaaaleqabaGaey4fIOcaaaGccaGL7bGaayzFaaGaeiilaWcaaiaaxMaacaWLjaWaaeWaaeaacqaIXaqmcqaIXaqmaiaawIcacaGLPaaaaaa@F92E@

where the expectation in the last equation is with respect to the conditional probability Pr^*^(*H*_*u *_= *h*|*D*_*u*_, *G*_*u*_, *X*_*u*_; θ^*^) ≡ *ρ*_*hu*_(θ^*^) that is defined as

ρhu(θ∗)=I{h∈S(Gu)}Pr⁡∗(Du,H=h|Xu;θ∗)∑h′∈S(Gu)Pr⁡∗(Du,H=h′|Xu;θ∗)=I{h∈S(Gu)}exp⁡{Du(βh+X′βhX)+m(h,X;γ)}∑h′∈S(Gu)exp⁡{Du(βh′+X′βh′X)+m(h′,X;γ)}=Pr⁡(Hu=h|Gu,Du,Xu;θ).     (12)
 MathType@MTEF@5@5@+=feaafiart1ev1aaatCvAUfKttLearuWrP9MDH5MBPbIqV92AaeXatLxBI9gBamrtHrhAL1wy0L2yHvtyaeHbnfgDOvwBHrxAJfwnaebbnrfifHhDYfgasaacH8akY=wiFfYdH8Gipec8Eeeu0xXdbba9frFj0=OqFfea0dXdd9vqai=hGuQ8kuc9pgc9s8qqaq=dirpe0xb9q8qiLsFr0=vr0=vr0dc8meaabaqaciaacaGaaeqabaWaaeGaeaaakeaafaqabeWadaaabaacciGae8xWdi3aaSbaaSqaaiabdIgaOjabdwha1bqabaGccqGGOaakcqWF4oqCdaahaaWcbeqaaiabgEHiQaaakiabcMcaPaqaaiabg2da9aqaamaalaaabaGaemysaK0aaiWaaeaacqWGObaAcqGHiiIZimaaliab+jr8tPGaeiikaGIaem4raC0aaSbaaSqaaiabdwha1bqabaGccqGGPaqkaiaawUhacaGL9baacyGGqbaucqGGYbGCdaahaaWcbeqaaiabgEHiQaaakiabcIcaOiabdseaenaaBaaaleaacqWG1bqDaeqaaOGaeiilaWIaemisaGKaeyypa0JaemiAaGMaeiiFaWNaemiwaG1aaSbaaSqaaiabdwha1bqabaGccqGG7aWocqWF4oqCdaahaaWcbeqaaiabgEHiQaaakiabcMcaPaqaamaaqababaGagiiuaaLaeiOCai3aaWbaaSqabeaacqGHxiIkaaaabaGafmiAaGMbauaacqGHiiIZcqGFse=ucqGGOaakcqWGhbWrdaWgaaadbaGaemyDauhabeaaliabcMcaPaqab0GaeyyeIuoakiabcIcaOiabdseaenaaBaaaleaacqWG1bqDaeqaaOGaeiilaWIaemisaGKaeyypa0JafmiAaGMbauaacqGG8baFcqWGybawdaWgaaWcbaGaemyDauhabeaakiabcUda7iab=H7aXnaaCaaaleqabaGaey4fIOcaaOGaeiykaKcaaaqaaaqaaiabg2da9aqaamaalaaabaGaemysaK0aaiWaaeaacqWGObaAcqGHiiIZliab+jr8tPGaeiikaGIaem4raC0aaSbaaSqaaiabdwha1bqabaGccqGGPaqkaiaawUhacaGL9baacyGGLbqzcqGG4baEcqGGWbaCdaGadaqaaiabdseaenaaBaaaleaacqWG1bqDaeqaaOGaeiikaGIae8NSdi2aaSbaaSqaaiabdIgaObqabaGccqGHRaWkcuWGybawgaqbaGGaaiab9j7aInaaBaaaleaacqWGObaAcqWGybawaeqaaOGaeiykaKIaey4kaSIaemyBa0MaeiikaGIaemiAaGMaeiilaWIaemiwaGLaei4oaSJae83SdCMaeiykaKcacaGL7bGaayzFaaaabaWaaabeaeaacyGGLbqzcqGG4baEcqGGWbaCdaGadaqaaiabdseaenaaBaaaleaacqWG1bqDaeqaaOGaeiikaGIae8NSdi2aaSbaaSqaaiqbdIgaOzaafaaabeaakiabgUcaRiqbdIfayzaafaGae8NSdi2aaSbaaSqaaiqbdIgaOzaafaGaemiwaGfabeaakiabcMcaPiabgUcaRiabd2gaTjabcIcaOiqbdIgaOzaafaGaeiilaWIaemiwaGLaei4oaSJae83SdCMaeiykaKcacaGL7bGaayzFaaaaleaacuWGObaAgaqbaiabgIGiolab+jr8tjabcIcaOiabdEeahnaaBaaameaacqWG1bqDaeqaaSGaeiykaKcabeqdcqGHris5aaaaaOqaaaqaaiabg2da9aqaaiGbccfaqjabckhaYjabcIcaOiabdIeainaaBaaaleaacqWG1bqDaeqaaOGaeyypa0JaemiAaGMaeiiFaWNaem4raC0aaSbaaSqaaiabdwha1bqabaGccqGGSaalcqWGebardaWgaaWcbaGaemyDauhabeaakiabcYcaSiabdIfaynaaBaaaleaacqWG1bqDaeqaaOGaei4oaSJae8hUdeNaeiykaKIaeiOla4caaiaaxMaacaWLjaWaaeWaaeaacqaIXaqmcqaIYaGmaiaawIcacaGLPaaaaaa@F659@

Note that with the proposed model Pr(*H *= *h*|*D*, *G*, *X*; *θ*) does not depend on *α*, hence can be readily evaluated even if *α *cannot be reliably estimated, which is usually the case in case-control studies. This property is not shared by other modeling strategies such as those in Spinka et al. [9] and Zhao [12], unless the rare-disease assumption is made.

Equation (11) suggests that the maximum likelihood estimate of θ^* ^can be obtained by the following EM algorithm. Given the estimate of θ^* ^from the *r*th iteration, denoted by θ^
 MathType@MTEF@5@5@+=feaafiart1ev1aaatCvAUfKttLearuWrP9MDH5MBPbIqV92AaeXatLxBI9gBaebbnrfifHhDYfgasaacH8akY=wiFfYdH8Gipec8Eeeu0xXdbba9frFj0=OqFfea0dXdd9vqai=hGuQ8kuc9pgc9s8qqaq=dirpe0xb9q8qiLsFr0=vr0=vr0dc8meaabaqaciaacaGaaeqabaqabeGadaaakeaaiiGacuWF4oqCgaqcaaaa@2E79@^*(*r*)^, we first evaluate the posterior haplotype-pair distribution *ρ*_*hu*_(θ^*^) at θ^* ^= θ^
 MathType@MTEF@5@5@+=feaafiart1ev1aaatCvAUfKttLearuWrP9MDH5MBPbIqV92AaeXatLxBI9gBaebbnrfifHhDYfgasaacH8akY=wiFfYdH8Gipec8Eeeu0xXdbba9frFj0=OqFfea0dXdd9vqai=hGuQ8kuc9pgc9s8qqaq=dirpe0xb9q8qiLsFr0=vr0=vr0dc8meaabaqaciaacaGaaeqabaqabeGadaaakeaaiiGacuWF4oqCgaqcaaaa@2E79@^*(*r*)^. Then we obtain the updated estimate θ^
 MathType@MTEF@5@5@+=feaafiart1ev1aaatCvAUfKttLearuWrP9MDH5MBPbIqV92AaeXatLxBI9gBaebbnrfifHhDYfgasaacH8akY=wiFfYdH8Gipec8Eeeu0xXdbba9frFj0=OqFfea0dXdd9vqai=hGuQ8kuc9pgc9s8qqaq=dirpe0xb9q8qiLsFr0=vr0=vr0dc8meaabaqaciaacaGaaeqabaqabeGadaaakeaaiiGacuWF4oqCgaqcaaaa@2E79@^*(*r*+1) ^by solving θ^* ^from

∑u=1N∑h=0M−1ρhuΨuC(θ∗)=0,     (13)
 MathType@MTEF@5@5@+=feaafiart1ev1aaatCvAUfKttLearuWrP9MDH5MBPbIqV92AaeXatLxBI9gBaebbnrfifHhDYfgasaacH8akY=wiFfYdH8Gipec8Eeeu0xXdbba9frFj0=OqFfea0dXdd9vqai=hGuQ8kuc9pgc9s8qqaq=dirpe0xb9q8qiLsFr0=vr0=vr0dc8meaabaqaciaacaGaaeqabaqabeGadaaakeaadaaeWbqaamaaqahabaacciGae8xWdi3aaSbaaSqaaiabdIgaOjabdwha1bqabaaabaGaemiAaGMaeyypa0JaeGimaadabaGaemyta0KaeyOeI0IaeGymaedaniabggHiLdGccqqHOoqwdaqhaaWcbaGaemyDauhabaGaem4qameaaaqaaiabdwha1jabg2da9iabigdaXaqaaiabd6eaobqdcqGHris5aOGaeiikaGIae8hUde3aaWbaaSqabeaacqGHxiIkaaGccqGGPaqkcqGH9aqpcqaIWaamcqGGSaalcaWLjaGaaCzcamaabmGabaGaeGymaeJaeG4mamdacaGLOaGaayzkaaaaaa@5116@

where *ρ*_*hu *_= *ρ*_*hu *_(θ^*(*r*)^). The process is repeated until some convergence criterion is met. Thus, with the proposed EM algorithm, maximum likelihood estimation with unphased genotype data under the proposed model can be readily implemented by iteratively fitting a weighted multinomial logistic regression model, with the iterative-updated weights *ρ*_*hu*_(θ^*^) given by (12). It can be seen that the EM algorithm above can accommodate not only the unphased genotype but also the missing genotype data.

When solving (13), we apply the Newton-Raphson algorithm with the negative derivative of ∑_*u *_∑_*h *_*ρ*_*hu *_ΨuC
 MathType@MTEF@5@5@+=feaafiart1ev1aaatCvAUfKttLearuWrP9MDH5MBPbIqV92AaeXatLxBI9gBaebbnrfifHhDYfgasaacH8akY=wiFfYdH8Gipec8Eeeu0xXdbba9frFj0=OqFfea0dXdd9vqai=hGuQ8kuc9pgc9s8qqaq=dirpe0xb9q8qiLsFr0=vr0=vr0dc8meaabaqaciaacaGaaeqabaqabeGadaaakeaacqqHOoqwdaqhaaWcbaGaemyDauhabaGaem4qameaaaaa@30EA@(θ^*^) with respective to θ^* ^approximated by the simple positive-definite matrix

ℐC=∑u=1NV∗{∂ΛDH(X;θ∗)∂θ∗|X=Xu},
 MathType@MTEF@5@5@+=feaafiart1ev1aaatCvAUfKttLearuWrP9MDH5MBPbIqV92AaeXatLxBI9gBamrtHrhAL1wy0L2yHvtyaeHbnfgDOvwBHrxAJfwnaebbnrfifHhDYfgasaacH8akY=wiFfYdH8Gipec8Eeeu0xXdbba9frFj0=OqFfea0dXdd9vqai=hGuQ8kuc9pgc9s8qqaq=dirpe0xb9q8qiLsFr0=vr0=vr0dc8meaabaqaciaacaGaaeqabaWaaeGaeaaakeaaimaacqWFqessdaahaaWcbeqaaGqaciab+neadbaakiabg2da9maaqahabaGaemOvay1aaWbaaSqabeaacqGHxiIkaaaabaGaemyDauNaeyypa0JaeGymaedabaGaemOta4eaniabggHiLdGcdaGadaqaamaalaaabaGaeyOaIyRaeu4MdW0aaSbaaSqaaiabdseaejabdIeaibqabaGccqGGOaakcqWGybawcqGG7aWoiiGacqqF4oqCdaahaaWcbeqaaiabgEHiQaaakiabcMcaPaqaaiabgkGi2kab9H7aXnaaCaaaleqabaGaey4fIOcaaaaakiabcYha8jabdIfayjabg2da9iabdIfaynaaBaaaleaacqWG1bqDaeqaaaGccaGL7bGaayzFaaGaeiilaWcaaa@5CDD@

where V^*^(·|*X*) denotes the variance with respect to Pr^*^(*D*, *H*|*X*; θ^*^); see Appendix 3 for the derivation. Note that ℐ
 MathType@MTEF@5@5@+=feaafiart1ev1aaatCvAUfKttLearuWrP9MDH5MBPbIqV92AaeXatLxBI9gBamrtHrhAL1wy0L2yHvtyaeHbnfgDOvwBHrxAJfwnaebbnrfifHhDYfgasaacH8akY=wiFfYdH8Gipec8Eeeu0xXdbba9frFj0=OqFfea0dXdd9vqai=hGuQ8kuc9pgc9s8qqaq=dirpe0xb9q8qiLsFr0=vr0=vr0dc8meaabaqaciaacaGaaeqabaWaaeGaeaaakeaaimaacqWFqessaaa@3769@^*C *^is also an approximation for the "complete-data" information matrix based on Pr^*^(*D*, *H*|*X*; θ^*^).

Let θ^
 MathType@MTEF@5@5@+=feaafiart1ev1aaatCvAUfKttLearuWrP9MDH5MBPbIqV92AaeXatLxBI9gBaebbnrfifHhDYfgasaacH8akY=wiFfYdH8Gipec8Eeeu0xXdbba9frFj0=OqFfea0dXdd9vqai=hGuQ8kuc9pgc9s8qqaq=dirpe0xb9q8qiLsFr0=vr0=vr0dc8meaabaqaciaacaGaaeqabaqabeGadaaakeaaiiGacuWF4oqCgaqcaaaa@2E79@^* ^be the final estimate given by the EM algorithm, provided convergence is achieved. Then θ^
 MathType@MTEF@5@5@+=feaafiart1ev1aaatCvAUfKttLearuWrP9MDH5MBPbIqV92AaeXatLxBI9gBaebbnrfifHhDYfgasaacH8akY=wiFfYdH8Gipec8Eeeu0xXdbba9frFj0=OqFfea0dXdd9vqai=hGuQ8kuc9pgc9s8qqaq=dirpe0xb9q8qiLsFr0=vr0=vr0dc8meaabaqaciaacaGaaeqabaqabeGadaaakeaaiiGacuWF4oqCgaqcaaaa@2E79@^* ^is the maximum likelihood estimate of θ^*^, which is asymptotically normal. The covariance matrix for the maximum likelihood estimates of all the parameters, except the intercept parameter α^*^, can be estimated by the corresponding submatrix of the inverse of the observed information matrix based on *L*_*P*_. Following Louis [16], the observed information matrix based on *L*_*P *_can be obtained by

ℐC−∑u=1N{E(ΨuCΦuC′|Du,GuXu)−ΨuΨ′u}
 MathType@MTEF@5@5@+=feaafiart1ev1aaatCvAUfKttLearuWrP9MDH5MBPbIqV92AaeXatLxBI9gBamrtHrhAL1wy0L2yHvtyaeHbnfgDOvwBHrxAJfwnaebbnrfifHhDYfgasaacH8akY=wiFfYdH8Gipec8Eeeu0xXdbba9frFj0=OqFfea0dXdd9vqai=hGuQ8kuc9pgc9s8qqaq=dirpe0xb9q8qiLsFr0=vr0=vr0dc8meaabaqaciaacaGaaeqabaWaaeGaeaaakeaaimaacqWFqessdaahaaWcbeqaaGqaciab+neadbaakiabgkHiTmaaqahabaWaaiWaaeaacqWGfbqrcqGGOaakcqqHOoqwdaqhaaWcbaGaemyDauhabaGaem4qameaaOGaeuOPdy0aa0baaSqaaiabdwha1bqaaiqbdoeadzaafaaaaOGaeiiFaWNaemiraq0aaSbaaSqaaiabdwha1bqabaGccqGGSaalcqWGhbWrdaWgaaWcbaGaemyDauhabeaakiabdIfaynaaBaaaleaacqWG1bqDaeqaaOGaeiykaKIaeyOeI0IaeuiQdK1aaSbaaSqaaiabdwha1bqabaGccuqHOoqwgaqbamaaBaaaleaacqWG1bqDaeqaaaGccaGL7bGaayzFaaaaleaacqWG1bqDcqGH9aqpcqaIXaqmaeaacqWGobGta0GaeyyeIuoaaaa@6014@

evaluated at θ^* ^= θ^
 MathType@MTEF@5@5@+=feaafiart1ev1aaatCvAUfKttLearuWrP9MDH5MBPbIqV92AaeXatLxBI9gBaebbnrfifHhDYfgasaacH8akY=wiFfYdH8Gipec8Eeeu0xXdbba9frFj0=OqFfea0dXdd9vqai=hGuQ8kuc9pgc9s8qqaq=dirpe0xb9q8qiLsFr0=vr0=vr0dc8meaabaqaciaacaGaaeqabaqabeGadaaakeaaiiGacuWF4oqCgaqcaaaa@2E79@^*^.

The EM algorithm described above is found to be sensitive to the initial estimate of *γ*, the parameter associated with the haplotype-pair distribution in the control population, in that the algorithm may fail to converge when using inappropriate initial estimates of *γ*. To obtain an adequate initial estimate of *γ*, we can adopt an approach similar to the proposal in Zhao et al. [12] to obtain the initial estimate for *γ *based on the control data only. For example, when the haplotype distribution in the controls follows HWE and is independent of environmental covariates, we can obtain initial estimate for *γ*, or equivalently the haplotype frequencies *π *in the controls, by solving *π*_*j*_, *j *= 0,..., *J *- 1 from

0=∑u=1N∑h∈S(Gu)(1−Du)ρ˜hu(π(−1)){I(h1=ℏj)+I(h2=ℏj)−2πj},j=0,...,J−1,     (14)
 MathType@MTEF@5@5@+=feaafiart1ev1aaatCvAUfKttLearuWrP9MDH5MBPbIqV92AaeXatLxBI9gBamrtHrhAL1wy0L2yHvtyaeHbnfgDOvwBHrxAJfwnaebbnrfifHhDYfgasaacH8akY=wiFfYdH8Gipec8Eeeu0xXdbba9frFj0=OqFfea0dXdd9vqai=hGuQ8kuc9pgc9s8qqaq=dirpe0xb9q8qiLsFr0=vr0=vr0dc8meaabaqaciaacaGaaeqabaWaaeGaeaaakeaafaqabeqacaaabaGaeGimaaJaeyypa0ZaaabCaeaadaaeqbqaaiabcIcaOiabigdaXiabgkHiTiabdseaenaaBaaaleaacqWG1bqDaeqaaOGaeiykaKccciGaf8xWdiNbaGaadaWgaaWcbaGaemiAaGMaemyDauhabeaaaeaacqWGObaAcqGHiiIZimaacqGFse=ucqGGOaakcqWGhbWrdaWgaaadbaGaemyDauhabeaaliabcMcaPaqab0GaeyyeIuoakiabcIcaOiab=b8aWnaaCaaaleqabaGaeiikaGIaeyOeI0IaeGymaeJaeiykaKcaaOGaeiykaKYaaiWaaeaacqWGjbqscqGGOaakcqWGObaAdaahaaWcbeqaaiabigdaXaaakiabg2da9iabl+qiOnaaBaaaleaacqWGQbGAaeqaaOGaeiykaKIaey4kaSIaemysaKKaeiikaGIaemiAaG2aaWbaaSqabeaacqaIYaGmaaGccqGH9aqpcqWIpecAdaWgaaWcbaGaemOAaOgabeaakiabcMcaPiabgkHiTiabikdaYiab=b8aWnaaBaaaleaacqWGQbGAaeqaaaGccaGL7bGaayzFaaGaeiilaWcaleaacqWG1bqDcqGH9aqpcqaIXaqmaeaacqWGobGta0GaeyyeIuoaaOqaaiabdQgaQjabg2da9iabicdaWiabcYcaSiabc6caUiabc6caUiabc6caUiabcYcaSiabdQeakjabgkHiTiabigdaXaaacqGGSaalcaWLjaGaaCzcamaabmaabaGaeGymaeJaeGinaqdacaGLOaGaayzkaaaaaa@8860@

where

ρ˜hu(π(−1))=Pr⁡∗(H=h|Du=0,Gu;π(−1))=I{h∈S(Gu)}πh1(−1)πh2(−1)∑hI{h∈S(Gu)}πh1(−1)πh2(−1), h=0,...,M−1,
 MathType@MTEF@5@5@+=feaafiart1ev1aaatCvAUfKttLearuWrP9MDH5MBPbIqV92AaeXatLxBI9gBamrtHrhAL1wy0L2yHvtyaeHbnfgDOvwBHrxAJfwnaebbnrfifHhDYfgasaacH8akY=wiFfYdH8Gipec8Eeeu0xXdbba9frFj0=OqFfea0dXdd9vqai=hGuQ8kuc9pgc9s8qqaq=dirpe0xb9q8qiLsFr0=vr0=vr0dc8meaabaqaciaacaGaaeqabaWaaeGaeaaakeaafaqadeGabaaabaacciGaf8xWdiNbaGaadaWgaaWcbaGaemiAaGMaemyDauhabeaakiabcIcaOiabec8aWnaaCaaaleqabaGaeiikaGIaeyOeI0IaeGymaeJaeiykaKcaaOGaeiykaKIaeyypa0JagiiuaaLaeiOCai3aaWbaaSqabeaacqGHxiIkaaGccqGGOaakcqWGibascqGH9aqpcqWGObaAcqGG8baFcqWGebardaWgaaWcbaGaemyDauhabeaakiabg2da9iabicdaWiabcYcaSiabdEeahnaaBaaaleaacqWG1bqDaeqaaOGaei4oaSJae8hWda3aaWbaaSqabeaacqGGOaakcqGHsislcqaIXaqmcqGGPaqkaaGccqGGPaqkaeaacqGH9aqpdaWcaaqaaiabdMeajnaacmaabaGaemiAaGMaeyicI4mcdaWccqGFse=ukiabcIcaOiabdEeahnaaBaaaleaacqWG1bqDaeqaaOGaeiykaKcacaGL7bGaayzFaaGae8hWda3aa0baaSqaaiabdIgaOnaaCaaameqabaGaeGymaedaaaWcbaGaeiikaGIaeyOeI0IaeGymaeJaeiykaKcaaOGae8hWda3aa0baaSqaaiabdIgaOnaaCaaameqabaGaeGOmaidaaaWcbaGaeiikaGIaeyOeI0IaeGymaeJaeiykaKcaaaGcbaWaaabeaeaacqWGjbqsdaGadaqaaiabdIgaOjabgIGioVGae4NeXpLccqGGOaakcqWGhbWrdaWgaaWcbaGaemyDauhabeaakiabcMcaPaGaay5Eaiaaw2haaiab=b8aWnaaDaaaleaacqWGObaAdaahaaadbeqaaiabigdaXaaaaSqaaiabcIcaOiabgkHiTiabigdaXiabcMcaPaaakiab=b8aWnaaDaaaleaacqWGObaAdaahaaadbeqaaiabikdaYaaaaSqaaiabcIcaOiabgkHiTiabigdaXiabcMcaPaaaaeaacqWGObaAaeqaniabggHiLdaaaOGaeiilaWIaeeiiaaIaemiAaGMaeyypa0JaeGimaaJaeiilaWIaeiOla4IaeiOla4IaeiOla4IaeiilaWIaemyta0KaeyOeI0IaeGymaeJaeiilaWcaaaaa@A75B@

and *π*^(-1) ^denotes the solution of *π *in the previous iteration. Our numerical experiments reveal that, starting with assigning equal frequency to each haplotype, two iterations in (14) are sufficient to yield an adequate initial estimate of *γ*.

To ensure stable estimation during the EM algorithm, for a haplotype with very small estimated frequency (e.g. <*e*^-10^), we will fix its frequency to be 0 and drop the corresponding parameter from *γ*.

We have developed a general-purposed SAS Macro for implementing the proposed method, which is available upon request from the corresponding author.

## Results

### Application to the hypertriglyceridemia study

The proposed methodology is applied to the hypertriglyceridemia study conducted at National Taiwan University Hospital (Kao el al. [17], Tzeng et al. [18]), where 303 healthy controls and 290 cases, defined as serum triglyceride level > 400 mg/dl, were recruited. One primary objective of this study is to assess the association between the haplotypes in apolipoprotein A5 gene (APOA5) and hypertriglyceridemia in humans, adjusting for the environmental covariates Age, Sex, and BMI, and to explore the potential haplotype-environment interactions.

Based on the control genotype data for 5 polymorphic sites in APOA5 (IVS3+476, c.457, c.553, c.1177, c.1250), 6 common haplotypes are derived by the EM algorithm, including GGGCT (66.8%), AGGCC (15.3%), GGTCT (4.2%), GAGTT (9.5%), AGGCT (1.2%), GGGTT (0.9%), which are labeled as *ħ*_*j*_, *j *= 0,..., 5, and the most frequent haplotype *ħ*_0 _= GGGCT is chosen as the reference haplotype. We then fit a multinomial logistlic model, where the disease risk model (8) is specified by *β*_*h *_= Z′h
 MathType@MTEF@5@5@+=feaafiart1ev1aaatCvAUfKttLearuWrP9MDH5MBPbIqV92AaeXatLxBI9gBaebbnrfifHhDYfgasaacH8akY=wiFfYdH8Gipec8Eeeu0xXdbba9frFj0=OqFfea0dXdd9vqai=hGuQ8kuc9pgc9s8qqaq=dirpe0xb9q8qiLsFr0=vr0=vr0dc8meaabaqaciaacaGaaeqabaqabeGadaaakeaacuWGAbGwgaqbamaaBaaaleaacqWGObaAaeqaaaaa@2F7A@*β*_*hap *_with *Z*_*h *_= {*I*(*h*^1 ^= *ħ*_*j*_) + *I *(*h*^2 ^= *ħ*_*j*_), *j = *1,..., 5}, and the environmental covariates *X *including Age, Sex, and BMI. The haplotype-environment interaction terms include only the interactions of the haplotype GGTCT with Age and BMI, since these are the only promising interactions according to preliminary analysis. The model (5) for the haplotype-pair distribution in the controls is specified by *m*(*h*, *X*; *γ*) = *γ*_0*h *_+ *γ*_1*h *_*S*, where *γ*_0*h *_= Z′h
 MathType@MTEF@5@5@+=feaafiart1ev1aaatCvAUfKttLearuWrP9MDH5MBPbIqV92AaeXatLxBI9gBaebbnrfifHhDYfgasaacH8akY=wiFfYdH8Gipec8Eeeu0xXdbba9frFj0=OqFfea0dXdd9vqai=hGuQ8kuc9pgc9s8qqaq=dirpe0xb9q8qiLsFr0=vr0=vr0dc8meaabaqaciaacaGaaeqabaqabeGadaaakeaacuWGAbGwgaqbamaaBaaaleaacqWGObaAaeqaaaaa@2F7A@*γ*_0 _and *γ*_1*h *_= Z′h
 MathType@MTEF@5@5@+=feaafiart1ev1aaatCvAUfKttLearuWrP9MDH5MBPbIqV92AaeXatLxBI9gBaebbnrfifHhDYfgasaacH8akY=wiFfYdH8Gipec8Eeeu0xXdbba9frFj0=OqFfea0dXdd9vqai=hGuQ8kuc9pgc9s8qqaq=dirpe0xb9q8qiLsFr0=vr0=vr0dc8meaabaqaciaacaGaaeqabaqabeGadaaakeaacuWGAbGwgaqbamaaBaaaleaacqWGObaAaeqaaaaa@2F7A@*γ*_1 _with *Z*_*h *_defined as above, and *S *= *I*(BMI > 23.2), the indicator of BMI being larger than its mean in the controls. By this model we allow the dependence between BMI and haplotypes; the dependence between other environmental factors (Age, Sex) and haplotypes is less significant in light of preliminary analysis, and hence is not considered in the final analysis.

The analysis results are displayed in Table 1. Relative to the common haplotype GGGCT, the three hapiotypes AGGCC, GGTCT, and GAGTT are associated with higher risk of hypertriglyceridemia; the former two were also identified elsewhere by haplotype-specific analysis (Pennacchio et al. [19], Kao et al. [17]), and here by joint analysis of multiple haplotype effects we further identify GAGTT as a potential risk haplotype. The significant interaction term suggests that the effect associated with the haplotype GGTCT is modified by age: older carriers of the GGTCT haplotype have decreased risk for hypertriglyceridemia than younger carriers. The estimates of *γ*_1 _(data not shown) reveal that BMI and the haplotypes GGTCT and GGGCT are dependent in the control population.

**Table 1:** Analysis results of the hypertriglyceridemia data

variable	coefficient estimate	SE	P-value
GGGCT (reference)	0	-	-
AGGCC	1.533	0.187	< 0.0001
GGTCT	2.766	0.255	< 0.0001
GAGTT	0.994	0.260	0.0001
AGGCT	1.140	0.584	0.051
GGGTT	0.606	0.649	0.351
GGTCT*Age	-0.020	0.010	0.044
GGTCT*BMI	-0.052	0.040	0.194
Age (years)	0.030	0.010	0.003
Sex (female)	-0.263	0.210	0.212
BMI (kg/m^2^)	0.293	0.040	< 0.0001

### Simulation studies

The first simulation study is to examine the finite sample performance of the proposed method. In each replication, data on the environmental variable *X *are simulated from a standard normal distribution. Given *X*, the haplotype-pair distribution in the control population is assumed to be Pr(*H*^1 ^= *ħ*_*j*_, *H*^2 ^= *ħ*_*k*_|*X*, *D *= 0) = Pr(*ħ*_*j*_|*S*, *D *= 0) Pr(*ħ*_*k*_|*S*, *D *= 0) with *S *= *I*(*X *> 0), namely the distribution of *H *follows HWE within each of the strata defined by whether *X *> 0 or not. This specification corresponds to a situation where the hapiotypes and environment covariates are dependent and hence the gene-environment independence assumption does not hold in the control population; see Chatterjee and Carroll [8] for some examples where gene and environmental factors may be dependent. Following Satten and Epstein [14], we assume the haplotypes contain 5 tightly-linked SNPs, and the associated haplotype frequencies {Pr(*ħ*_*j*_|*S*,*D *= 0), *j *= 0,..., *J*-1} in stratum *S *are listed in Table 2 for *S *= 0, 1. By convention, we choose the most common haplotype *ħ*_0 _= "10011" as the reference haplotype. Further, we choose the haplotype *ħ*_4 _= "01100" as a putative susceptibility haplotype. Data on the haplotype pair and disease phenotype are then generated according to the multinomial logistic model (7), with *β*_*H *_and *β*_*HX *_specified respectively as *β*_*H *_= *β*_*hap*_*Z*_*H *_and *β*_*HX *_= *β*_*int*_*XZ*_*H*_, where we take *Z*_*H *_to be either *I*(*H*^1 ^= *ħ*_4_) *I*(*H*^2 ^= *ħ*_4_), *I*(*H*^1 ^= *ħ*_4_) + *I*(*H*^2 ^= *ħ*_4_) - *I*(*H*^1 ^= *ħ*_4_)*I*(*H*^2 ^= *ħ*_4_), or *I*(*H*^1 ^= *ħ*_4_) + *I*(*H*^2 ^= *ħ*_4_) when a recessive, dominant, or multiplicative genetic law is assumed, and *β*_*hap *_and *β*_*int *_are respectively the marginal effect of the risk haplotype *ħ*_4 _and the interaction between this haplotype and *X*. A case-control sample with 415 controls and 796 cases is then selected from a larger set of data. When analyzing the data, we ignore the phase information for haplotype data and use only the unphased genotype data. Further, we allow the genotype data to be missing independently and randomly for SNPs 1–5 with probabilities 2.9, 5.6, 5.4, 4.5, and 2.3%, respectively.

**Table 2:** Haplotypes and frequencies used in simulation studies

		frequencies Pr(*ħ*|*S*, *D *= 0)	
		
label	haplotype	*S *= 0	*S *= 1
*ħ*_0_	10011	0.3327	0.3624
*ħ*_1_	00100	0.0037	0.0040
*ħ*_2_	00110	0.0010	0.0011
*ħ*_3_	01011	0.1409	0.1535
*ħ*_4_	01100	0.2489	0.1818
*ħ*_5_	01101	0.0005	0.0005
*ħ*_6_	01110	0.0035	0.0038
*ħ*_7_	01111	0.0007	0.0008
*ħ*_8_	10000	0.0129	0.0140
*ħ*_9_	10010	0 0009	0.0010
*ħ*_10_	00010	0.0063	0.0069
*ħ*_11_	10100	0.0611	0.0666
*ħ*_12_	10110	0.0336	0.0366
*ħ*_13_	11011	0.1416	0.1542
*ħ*_14_	11100	0.0101	0.0110
*ħ*_15_	11110	0.0009	0.0010
*ħ*_16_	11111	0.0007	0.0008

In each setting considered, we set *α *= -3, *β*_*X *_= 0.3 and *β*_*hap *_= 0.1. The haplotype-environment interaction parameter *β*_*int*_, which is the main focus here, is set to 0, 0.15, or 0.3. The results based on 500 replications are displayed in Table 3. The point and standard error estimates for the association parameters are essentially unbiased, and the coverage of the 95% confidence intervals is close to the nominal value. In particular, the size of the Wald test for testing *H*_0 _: *β*_*int *_= 0 essentially attains its nominal value, and the associated power for detecting haplotype-environment interaction is satisfactory when *β*_*int *_= 0.3. The point and standard error estimates for the haplotype frequencies also match the true values well (results not shown). We also conduct analysis where the effects of rare haplotypes (with frequency 1%–5%) are estimated (data not shown). The point estimate essentially remains unbiased, while the standard-error estimate underestimates the true value. As commented by a referee, here a permutation-based procedure may be required for proper inference.

**Table 3:** Summary statistics for the first simulation studies

	Recessive Law			Dominant Law			Multiplicative Law		
	*β*_*X*_	*β*_*hap*_	*β*_*int*_	*β*_*x*_	*β*_*hap*_	*β*_*int*_	*β*_*x*_	*β*_*hap*_	*β*_*int*_
*β*_*x *_= 0.3, *β*_*hap *_= 0.1, *β*_*int *_= 0									
bias^*a*^	0.000	-0.030	-0.002	-0.001	-0.003	0.004	0.003	0.000	-0.007
*SE*^*b*^	0.064	0.200	0.186	0.072	0.121	0.094	0.071	0.112	0.082
SE_ MathType@MTEF@5@5@+=feaafiart1ev1aaatCvAUfKttLearuWrP9MDH5MBPbIqV92AaeXatLxBI9gBaebbnrfifHhDYfgasaacH8akY=wiFfYdH8Gipec8Eeeu0xXdbba9frFj0=OqFfea0dXdd9vqai=hGuQ8kuc9pgc9s8qqaq=dirpe0xb9q8qiLsFr0=vr0=vr0dc8meaabaqaciaacaGaaeqabaqabeGadaaakeGabaaQgmaaHaaabiqaaGQbcqqGtbWucqqGfbqraiaawkWaaaaa@310E@^*c*^	0.063	0.198	0.184	0.073	0.119	0.096	0.072	0.108	0.082
cover^*d*^	0.944	0.945	0.948	0.955	0.950	0.957	0.959	0.943	0.946
size^*e*^	-	-	0.053	-	-	0.043	-	-	0.054
*β*_*X *_= 0.3, *β*_*hap *_= 0.1, *β*_*int *_= 0.15									
bias^*a*^	0.002	0.031	-0.011	0.001	0.006	0.000	0.003	-0.003	0.008
SE^*b*^	0.061	0.196	0.183	0.075	0.120	0.099	0.075	0.104	0.085
SE_ MathType@MTEF@5@5@+=feaafiart1ev1aaatCvAUfKttLearuWrP9MDH5MBPbIqV92AaeXatLxBI9gBaebbnrfifHhDYfgasaacH8akY=wiFfYdH8Gipec8Eeeu0xXdbba9frFj0=OqFfea0dXdd9vqai=hGuQ8kuc9pgc9s8qqaq=dirpe0xb9q8qiLsFr0=vr0=vr0dc8meaabaqaciaacaGaaeqabaqabeGadaaakeGabaaQgmaaHaaabiqaaGQbcqqGtbWucqqGfbqraiaawkWaaaaa@310E@^*c*^	0.063	0.199	0.180	0.073	0.120	0.094	0.072	0.109	0.079
cover^*d*^	0.953	0.967	0.943	0.950	0.945	0.950	0.948	0.965	0.925
power^*f*^	-	-	0.155	-	-	0.390	-	-	0.518
*β*_*x *_= 0.3, *β*_*hap *_= 0.1, *β*_*int *_= 0.3									
bias^*a*^	0.000	-0.027	0.002	0.004	0.004	0.010	0.000	0.003	0.002
SE^*b*^	0.067	0.204	0.168	0.074	0.112	0.089	0.070	0.110	0.074
SE_ MathType@MTEF@5@5@+=feaafiart1ev1aaatCvAUfKttLearuWrP9MDH5MBPbIqV92AaeXatLxBI9gBaebbnrfifHhDYfgasaacH8akY=wiFfYdH8Gipec8Eeeu0xXdbba9frFj0=OqFfea0dXdd9vqai=hGuQ8kuc9pgc9s8qqaq=dirpe0xb9q8qiLsFr0=vr0=vr0dc8meaabaqaciaacaGaaeqabaqabeGadaaakeGabaaQgmaaHaaabiqaaGQbcqqGtbWucqqGfbqraiaawkWaaaaa@310E@^*c*^	0.063	0.201	0.174	0.074	0.121	0.093	0.072	0.111	0.076
cover^*d*^	0.933	0.965	0.957	0.952	0.967	0.952	0.967	0.947	0.960
power^*f*^	-	-	0.425	-	-	0.932	-	-	0.978

^*a*^Simulation mean of the parameter estimates minus the true value.

^*b*^Simulation standard error of the parameter estimates.

^*c*^Simulation mean of the estimated standard errors.

^*d*^Coverage probability of 95% confidence interval.

^*e*^Size of Wald test for testing *H*_0 _:*β*_*int *_= 0.

^*f*^Power of Wald test for testing *H*_0 _: *β*_*int *_= 0.

In the second simulation study, we compare the proposed method with some existing alternatives, including the methods by Zhao et al. [12] and by Spinka et al. [9]. To facilitate the comparison among these methods that are based on different assumptions, we conduct the simulation under a setting where the disease is rare, and the haplotype-pair distribution in the whole population follows HWE and is independent of the environmental covariate. All the methods considered can apply well under this setting. Specifically, in each simulation we first simulate the environmental covariate *X *from a standard normal distribution, and then simulate the haplotype pairs in the whole population according to HWE with the marginal haplotype frequencies given in the third column (*S *= 0) of Table 2. The disease phenotype is then generated by the logistic regression model

logit {Pr(*D*|*H*, *X*)} = *α *+ *β*_*H*_*H *+ *β*_*X*_*X *+ *β*_*HX*_*HX*,     (15)

where *β*_*H *_= *β*_*hap*_*Z*_*H*_, *β*_*HX *_= *β*_*int*_*Z*_*H*_*X *with *Z*_*H *_= *I*(*H*^1 ^= *ħ*_4_) *I*(*H*^2 ^= *ħ*_4_), and *α *= -3, *β*_*X *_= 0.3, *β*_*hap *_= 0.1, *β*_*int *_= 0 or 0.3. The phase information is ignored and only the unphased genotype information is used when analyzing the simulated data. We also allow the genotype data for SNPs 1–5 to be missing independently and randomly with probabilities as in the first simulation study.

When applying the methods of Zhao et al. [12] and Spinka et al. [9], we employ the same models used to generate the data, hence the model specification is fully correct. The method of Spinka et al. is implemented using either a grid-search method or the known value of Pr(*D *= 1) to estimate *α*. When applying our proposal, we simply assume the control haplotype-pair distribution is independent of *X *and satisfies HWE, which is in fact a moderately wrong specification in the current simulation setting.

Table 4 exhibits the simulation results based on 500 replications. Although applied with a moderate model mis-specification, the estimates from our proposal have small bias, and are more efficient than estimates from other approaches. The method of Spinka et al. [9] is less efficient than our proposal, even if the former further incorporates supplementary information on population disease prevalence. It is worth noting that, although the method of Spinka et al. and our proposal are both based on maximum likelihood estimation, they are in fact based on different modeling frameworks, hence they may results in parameter estimates with different efficiency. Both our proposal and the method of Spinka et al. are much more efficient than the method of Zhao et al. [12], consistent with the finding of Satten and Epstein [14] that fully prospective analysis such as the method of Zhao et al. may lose considerable efficiency for haplotype association analysis in case-control studies.

**Table 4:** Results of comparison of various methods, including Zhao et. al. [12] (Zhao), Spinka et al. [9] using grid-search (Spinka, grid) or supplementary information on Pr(*D *= 1) (Spinka, suppl.), and the proposed multinomial logistic regression method (Proposed)

	Zhao			Spinka, grid			Spinka, suppl.			Proposed		
				
	*β*_*X*_	*β*_*hap*_	*β*_*int*_	*β*_*X*_	*β*_*hap*_	*β*_*int*_	*β*_*X*_	*β*_*hap*_	*βint*	*β*_*X*_	*β*_*hap*_	*βint*
*α *= -3, *β*_*X *_= 0.3, *β*_*hap *_= 0.1, *β*_*int *_= 0												
bias^*a*^	0.002	0.025	0.028	0.002	-0.010	0.015	0.002	-0.020	-0.005	0.002	-0.021	-0.016
SE^*b*^	0.066	0.269	0.254	0.065	0.188	0.155	0.060	0.189	0.155	0.059	0.170	0.142
SE_ MathType@MTEF@5@5@+=feaafiart1ev1aaatCvAUfKttLearuWrP9MDH5MBPbIqV92AaeXatLxBI9gBaebbnrfifHhDYfgasaacH8akY=wiFfYdH8Gipec8Eeeu0xXdbba9frFj0=OqFfea0dXdd9vqai=hGuQ8kuc9pgc9s8qqaq=dirpe0xb9q8qiLsFr0=vr0=vr0dc8meaabaqaciaacaGaaeqabaqabeGadaaakeGabaaQgmaaHaaabiqaaGQbcqqGtbWucqqGfbqraiaawkWaaaaa@310E@^*c*^	0.064	0.260	0.268	0.063	0.187	0.159	0.063	0.183	0.153	0.063	0.175	0.143
cover^*d*^	0.948	0.958	0.975	0.955	0.957	0.965	0.943	0.947	0.952	0.970	0.947	0.955
size^*e*^	-	-	0.025	-	-	0.035	-	-	0.048	-	-	0.045
*α *= -3, *β*_*X *_= 0.3, *β*_*hap *_= 0.1,*β*_*int *_= 0.3												
bias^*a*^	0.001	0.022	0.029	0.000	-0.009	0.029	-0.006	-0.017	0.005	0.001	-0.028	-0.019
SE^*b*^	0.064	0.269	0.289	0.063	0.197	0.182	0.061	0.193	0.152	0.064	0.187	0.137
SE_ MathType@MTEF@5@5@+=feaafiart1ev1aaatCvAUfKttLearuWrP9MDH5MBPbIqV92AaeXatLxBI9gBaebbnrfifHhDYfgasaacH8akY=wiFfYdH8Gipec8Eeeu0xXdbba9frFj0=OqFfea0dXdd9vqai=hGuQ8kuc9pgc9s8qqaq=dirpe0xb9q8qiLsFr0=vr0=vr0dc8meaabaqaciaacaGaaeqabaqabeGadaaakeGabaaQgmaaHaaabiqaaGQbcqqGtbWucqqGfbqraiaawkWaaaaa@310E@^*c*^	0.064	0.265	0.277	0.063	0.193	0.161	0.063	0.189	0.154	0.063	0.181	0.137
cover^*d*^	0.950	0.952	0.936	0.948	0.958	0.923	0.954	0.948	0.950	0.954	0.948	0.956
power^*f*^	-	-	0.198	-	-	0.524	-	-	0.480	-	-	0.513

^*a*^Simulation mean of the parameter estimates minus the true value.

^*b*^Simulation standard error of the parameter estimates.

^*c*^Simulation mean of the estimated standard errors.

^*d*^Coverage probability of 95% confidence interval.

^*e*^Size of Wald test for testing *H*_0 _: *β*_*int *_= 0.

^*f*^Power of Wald test for testing *H*_0 _: *β*_*int *_= 0.

To examine the sensitivity of the proposed method to the model assumptions, we conduct a simulation study to assess the bias of the estimates when the haplotype-pair distribution is wrongly assumed to follow HWE in the proposed method. Specifically, here we generate the haplotype data in the controls from the model

Pr⁡(H1=ℏj,H2=ℏk|X,D=0)={(1−f)πjπkj≠k,fπj+(1−f)πj2j=k,
 MathType@MTEF@5@5@+=feaafiart1ev1aaatCvAUfKttLearuWrP9MDH5MBPbIqV92AaeXatLxBI9gBaebbnrfifHhDYfgasaacH8akY=wiFfYdH8Gipec8Eeeu0xXdbba9frFj0=OqFfea0dXdd9vqai=hGuQ8kuc9pgc9s8qqaq=dirpe0xb9q8qiLsFr0=vr0=vr0dc8meaabaqaciaacaGaaeqabaqabeGadaaakeaacyGGqbaucqGGYbGCcqGGOaakcqWGibasdaahaaWcbeqaaiabigdaXaaakiabg2da9iabl+qiOnaaBaaaleaacqWGQbGAaeqaaOGaeiilaWIaemisaG0aaWbaaSqabeaacqaIYaGmaaGccqGH9aqpcqWIpecAdaWgaaWcbaGaem4AaSgabeaakiabcYha8jabdIfayjabcYcaSiabdseaejabg2da9iabicdaWiabcMcaPiabg2da9maaceqabaqbaeaabiGaaaqaaiabcIcaOiabigdaXiabgkHiTiabdAgaMjabcMcaPGGaciab=b8aWnaaBaaaleaacqWGQbGAaeqaaOGae8hWda3aaSbaaSqaaiabdUgaRbqabaaakeaacqWGQbGAcqGHGjsUcqWGRbWAcqGGSaalaeaacqWGMbGzcqWFapaCdaWgaaWcbaGaemOAaOgabeaakiabgUcaRiabcIcaOiabigdaXiabgkHiTiabdAgaMjabcMcaPiab=b8aWnaaDaaaleaacqWGQbGAaeaacqaIYaGmaaaakeaacqWGQbGAcqGH9aqpcqWGRbWAcqGGSaalaaaacaGL7baaaaa@6ADB@

where the fixation index parameter *f *= 0.1 or 0.2, and *π*_*j*_, *j *= 0,...,16, are haplotype frequencies given in the third column (*S *= 0) of Table 2; namely, the control haplotype-pair distribution does not follow HWE. However, we wrongly assume that HWE holds for this distribution in our analysis model. The environmental covariate *X *is generated from standard normal distribution, and the disease phenotype is generated according to (15). From the results shown in Table 5, we observe remarkable biases for the estimates of the main haplotype effect *β*_*hap *_when the genetic law is recessive or dominant, though the estimates for the interaction parameter *β*_*int *_is rather robust. This observation is consistent with that made by Satten and Epstein [14]: the methods based on the retrospective likelihood, such as our proposal, is generally less robust to the model assumptions such as HWE.

**Table 5:** Summary statistics for the third simulation studies

	Recessive Law			Dominant Law			Multiplicative Law		
	*β*_*X*_	*β*_*hap*_	*β*_*int*_	*β*_*X*_	*β*_*hap*_	*β*_*int*_	*β*_*X*_	*β*_*hap*_	*β*_*int*_
*β*_*X *_= 0.3, *β*_*hap *_= 0.1, *β*_*int *_= 0									
fixation index *f *= 0.1									
bias	-0.002	0.410	0.003	0.004	0.182	0.004	-0.002	0.021	0.004
SE	0.066	0.146	0.124	0.070	0.118	0.074	0.072	0.115	0.060
SE_ MathType@MTEF@5@5@+=feaafiart1ev1aaatCvAUfKttLearuWrP9MDH5MBPbIqV92AaeXatLxBI9gBaebbnrfifHhDYfgasaacH8akY=wiFfYdH8Gipec8Eeeu0xXdbba9frFj0=OqFfea0dXdd9vqai=hGuQ8kuc9pgc9s8qqaq=dirpe0xb9q8qiLsFr0=vr0=vr0dc8meaabaqaciaacaGaaeqabaqabeGadaaakeGabaaQgmaaHaaabiqaaGQbcqqGtbWucqqGfbqraiaawkWaaaaa@310E@	0.063	0.163	0.126	0.070	0.114	0.075	0.070	0.102	0.058
cover	0.910	0.275	0.951	0.928	0.608	0.957	0.930	0.893	0.946
fixation index *f *= 0.2									
bias	0.001	0.775	0.003	0.006	0.370	-0.002	-0.005	0.026	0.005
SE	0.063	0.161	0.121	0.068	0.112	0.071	0.072	0.117	0.064
SE_ MathType@MTEF@5@5@+=feaafiart1ev1aaatCvAUfKttLearuWrP9MDH5MBPbIqV92AaeXatLxBI9gBaebbnrfifHhDYfgasaacH8akY=wiFfYdH8Gipec8Eeeu0xXdbba9frFj0=OqFfea0dXdd9vqai=hGuQ8kuc9pgc9s8qqaq=dirpe0xb9q8qiLsFr0=vr0=vr0dc8meaabaqaciaacaGaaeqabaqabeGadaaakeGabaaQgmaaHaaabiqaaGQbcqqGtbWucqqGfbqraiaawkWaaaaa@310E@	0.063	0.155	0.115	0.070	0.113	0.075	0.070	0.102	0.058
cover	0.928	0	0.925	0.928	0.105	0.956	0.910	0.873	0.922

## Discussion

In the absence of environmental covariates, Epstein and Satten [7] (see also Satten and Epstein [14]) constructed their likelihood using a fully retrospective parameterization based only on (5) and (6). Therefore, in the absence of environmental covariates, the retrospective likelihood in Epstein and Satten [7] is exactly equivalent to that considered in our proposal. Our proposal, though based on a retrospective likelihood, can be implemented as if it were a prospective likelihood by introducing a multinomial logistic model and applying the profile livelihood approach given in Methods section. This is a preferred feature since a prospective analysis is more straightforward than a retrospective one. Moreover, our proposal provides an important extension of the Epstein and Satten's method to further incorporate environmental covariates.

Recently, based on the work of Chatterjee and Carroll [8], Spinka et al. [9] developed a profile likelihood approach to genetic association analysis with missing genetic information. In particular, for haplotype association analysis with unphased genotype data, their approach is based on a prospective logistic disease model Pr(*D*|*H*, *X*) together with a parametric model for Pr(*H*|*X*), the haplotype-pair distribution in the whole population given the environmental covariates. The implementation of the method of Spinka et al. generally requires estimating the true intercept parameter *α *in the prospective disease model (8). Since in a case-control sample there would be little information on this parameter due to the nature of biased sampling, the information matrix would he nearly singular, causing computational problems. Spinka et al. [9] suggested using either a grid-search method or supplementary information on the population disease risk Pr(*D *= 1) to estimate *α*. They also suggested a rare-disease approximation thereby estimation of *α *is not needed. Note that an earlier proposal by Stram et al. [20] is equivalent to the method of Spinka et al. when there are no environmental covariates and the information on population disease prevalence is used.

Although both the two methods have a prospective logistic model Pr(*D*|*H*, *X*) for the disease risk, our proposal specifies a model for Pr(*H*|*X*, *D*) = 0), the distribution of haplotype pairs in the control population given the covariates, while the method of Spinka et al. specifies the whole-population counterpart Pr(*H*|*X*).

In practice, although the assumption of HWE in the whole population may usually imply the same assumption hold in the controls, on the contrary, HWE in the controls may not necessarily imply HWE in the whole population when the disease is not rare. Owing to the nature of biased sampling, in a case-control sample it would be more plausible to check distributional assumptions for the control population than for the whole population, since in case-control studies estimating the control distribution Pr(*H*|*X*, *D *= 0) is more straightforward than estimating the population distribution Pr(*H*|*X*). Consequently, the proposed modeling strategy seems more suited to the case-control design.

When the disease is rare, Spinka et al. [9] suggested a rare-disease approximation when the disease prevalence is unknown. Their approximated likelihood has the same form as our proposal. Note that the validity of our likelihood does not rely on any rare-disease assumption; it serves as an exact likelihood in its own right under the modeling setup we consider. This implies that, for a common disease with unknown disease prevalence, our proposal can still work well when a suitable model can be specified for the haplotype-pair distribution in the controls, while the proposal of Spinka et al. with the rare-disease approximation can result in severe bias in this case. To illustrate this, we conduct a simulation with the same setting as in the second simulation study described in the previous section, except that a is now set to -0.5, corresponding to a common-disease situation. In the case when (*β*_*X*_, *β*_*hap*_, *β*_*int*_) = (0.3, 0.1, 0.3), the estimates of Spinka et al. with rare-disease approximation have substantial biases (-0.005, -0.76, -0.168) for these parameters.

Zhao et al. [12] proposed an estimating equation approach to haplotype association analysis, which is based on the score of a prospective likelihood; a similar prospective-likelihood approach for haplotype-environment interaction is also proposed by Lake et al [21]. These approaches are very easy to implement, and are particularly suitable for the case where a larger number of SNPs are involved. Also, they are found to be quite robust to mis-specification of the haplotype-pair distribution. The main drawback for such approaches is that they may be remarkably inefficient as compared to the retrospective likelihood-based methods such as our proposal and the method of Spinka et al. [9]; see Satten and Epstein [14] for a comprehensive efficiency comparison.

## Conclusion

To assess the association of haplotype and environmental factors with disease, we have proposed a new modeling setup that can be integrated into a multinomial logistic model, and can also be decomposed into a prospective logistic model relating the haplotype and environmental factors with disease, as well as a parametric model for the haplotype-pair distribution in the control population given the environmental covariates. The new proposal amounts to a natural extension of the method of Epstein and Satten [7] to further incorporate environmental covariates. The modeling strategy we adopt is very suited to the case-control design in the sense that, in contrast to the procedure proposed by Spinka et al. [9], the maximum likelihood estimation for the proposed model does not require any information on the population disease risk, which is usually lacking in a case-control sample. In fact, the maximum likelihood estimation for the proposed model with case-control data can be readily performed by applying a typical EM algorithm to a prospective multinomial logistic regression model. A SAS Macro implementing the proposed method is available from the corresponding author.

Note that the proposed method does not rely on specific modeling assumptions such as rare-disease, gene-environmental independence, and Hardy-Weinberg equilibrium assumptions, hence is applicable in very general settings, as long as the models involved are identifiable and appropriately specified. In addition, simulation results show that our proposal can achieve satisfactory efficiency. Accordingly, our proposal may serve as a useful tool for assessing the haplotype-environment associations with disease in population-based case-control studies. One limitation is that, unlike the prospective analysis, the proposed method, which is based on a retrospective likelihood, is sensitive to the model assumptions; namely, model mis-specification may lead to substantial bias, as commented by Epstein and Satten [7]. Therefore, the model specification is crucial in the proposed method to warrant valid analysis.

Some additional work is needed to strengthen the utility of the proposed method. First, if the main interest is in the association parameters and the haplotype-pair distribution is regarded as nuisance, then it would be desirable to improve the proposed method so that it can still yield valid estimates for the association parameters while allowing the model for the haplotype-pair distribution to be slightly misspecified.

Another important issue for haplotype-based association analysis involves the variable selection. When dealing with a larger number of haplotypes, efficient and effective methodologies for variable selection is crucial for finding the haplotypes contributing to liability for complex diseases [24,1]. Promising strategies include the step-wise selection [22], Lasso [23,24] and the false discovery rate (FDR) procedure [25,24]. How to incorporate appropriate variable selection procedures in the proposed multinomial logistic model with unphased genotype data deserves further investigation.

## Authors' contributions

YHC contributed to the development of the statistical methodology, the conducting of the data analysis and the simulations, and the writing of the manuscript. JTK contributed to the design and management of the hypertriglyceridemia study, and helped explain the results of data analysis.

## Appendix

### Appendix 1 the identifiability conditions

Given a model for *m*(*H*, *X*; *γ*) (the haplotype-pair distribution in the control population given covariates) and a fixed value of *γ*, identifiability conditions of the models for *β*_*H *_(main effects of haplotype pairs) and *β*_*HX *_(haplotype-environment interactions) can be obtained by arguments similar to those in Epstein and Satten [7]. Let *β *be the collection of the parameters involved in *β*_*H *_and *β*_*HX*_. According to (9), for any covariate value *x *observed in the case sample, the value of Pr(*D *= 1, *G *= *g*|*X *= *x*;*θ*) remains unchanged for a change in *β *if there exists some vector *φ *such that *φ*'*R*_*g*_(*x*) = *c *for every genotype *g*, where *c *is a constant and

Rg(x)=(∂/∂β)[∑H∈S(g)exp⁡{D(βH+x′βHX)+m(H,x;γ)}].
 MathType@MTEF@5@5@+=feaafiart1ev1aaatCvAUfKttLearuWrP9MDH5MBPbIqV92AaeXatLxBI9gBamrtHrhAL1wy0L2yHvtyaeHbnfgDOvwBHrxAJfwnaebbnrfifHhDYfgasaacH8akY=wiFfYdH8Gipec8Eeeu0xXdbba9frFj0=OqFfea0dXdd9vqai=hGuQ8kuc9pgc9s8qqaq=dirpe0xb9q8qiLsFr0=vr0=vr0dc8meaabaqaciaacaGaaeqabaWaaeGaeaaakeaacqWGsbGudaWgaaWcbaGaem4zaCgabeaakiabcIcaOiabdIha4jabcMcaPiabg2da9iabcIcaOiabgkGi2kabc+caViabgkGi2IGaciab=j7aIjabcMcaPiabcUfaBnaaqafabaGagiyzauMaeiiEaGNaeiiCaa3aaiWaaeaacqWGebarcqGGOaakcqWFYoGydaWgaaWcbaGaemisaGeabeaakiabgUcaRiqbdIha4zaafaGae8NSdi2aaSbaaSqaaiabdIeaijabdIfaybqabaGccqGGPaqkcqGHRaWkcqWGTbqBcqGGOaakcqWGibascqGGSaalcqWG4baEcqGG7aWocqWFZoWzcqGGPaqkaiaawUhacaGL9baaaSqaaiabdIeaijabgIGioJWaaiab+jr8tjabcIcaOiabdEgaNjabcMcaPaqab0GaeyyeIuoakiabc2faDjabc6caUaaa@6E19@

Let *R*(*x*) be the matrix with the *g*th row given by *R*_*g*_(*x*)'. Therefore, the models *β*_*H *_and *β*_*HX *_are identifiable if for any covariate value *x *we have: (1) *R*(*x*)'*R*(*x*) has full rank so that *R*(*x*)*φ *≠ 0 for any *φ *≠ 0, and (2) *R*(*x*)'1 = 0 where 1 denote a vector of ones.

### Appendix 2 the derivation of the profile likelihood

Since we treat the distribution *P *of *X *nonparametrically, it is sufficient to assume that *P *is discrete and has support points {*x*_1_,...,*x*_*K*_}, the unique values of *X *that are observed in the case-control sample. Let *G *= 0,..., *Q *- 1 denote labels for the observed unphased genotypes in the sample with *G *= 0 denoting a reference genotype and *Q *the number of distinct genotypes. Let *δ*_*k *_= Pr(*x*_*k*_), *k *= 1,..., *K*, *n*_*igk *_the number of subjects with *D *= *i*, *G *= *g *and *X *= *x*_*k*_, and *P*_*igk*_(*θ*) = Pr(*D *= *i*, *G *= *g*|*X *= *x*_*k*_; *θ*), *i *= 0, 1, *g *= 0,..., *Q *- 1, and *k *= 1,..., *K*. The loglikelihood for the case-control sample is given by

L(θ,δ)∑ifknigklog⁡Pigk(θ)δk∑g′k′Pig′k′(θ)δk′.
 MathType@MTEF@5@5@+=feaafiart1ev1aaatCvAUfKttLearuWrP9MDH5MBPbIqV92AaeXatLxBI9gBaebbnrfifHhDYfgasaacH8akY=wiFfYdH8Gipec8Eeeu0xXdbba9frFj0=OqFfea0dXdd9vqai=hGuQ8kuc9pgc9s8qqaq=dirpe0xb9q8qiLsFr0=vr0=vr0dc8meaabaqaciaacaGaaeqabaqabeGadaaakeaacqWGmbatcqGGOaakiiGacqWF4oqCcqGGSaalcqWF0oazcqGGPaqkdaaeqbqaaiabd6gaUnaaBaaaleaacqWGPbqAcqWGNbWzcqWGRbWAaeqaaaqaaiabdMgaPjabdAgaMjabdUgaRbqab0GaeyyeIuoakiGbcYgaSjabc+gaVjabcEgaNnaalaaabaGaemiuaa1aaSbaaSqaaiabdMgaPjabdEgaNjabdUgaRbqabaGccqGGOaakcqWF4oqCcqGGPaqkcqWF0oazdaWgaaWcbaGaem4AaSgabeaaaOqaamaaqababaGaemiuaa1aaSbaaSqaaiabdMgaPjqbdEgaNzaafaGafm4AaSMbauaaaeqaaaqaaiqbdEgaNzaafaGafm4AaSMbauaaaeqaniabggHiLdGccqGGOaakcqWF4oqCcqGGPaqkcqWF0oazdaWgaaWcbaGafm4AaSMbauaaaeqaaaaakiabc6caUaaa@617B@

The maximum likelihood estimate of *δ *for fixed *θ*, subject to ∑_*k *_*δ*_*k *_= 1, satisfies

δk=n++kN∑igνiPigk(θ), k=1,...,K,     (16)
 MathType@MTEF@5@5@+=feaafiart1ev1aaatCvAUfKttLearuWrP9MDH5MBPbIqV92AaeXatLxBI9gBaebbnrfifHhDYfgasaacH8akY=wiFfYdH8Gipec8Eeeu0xXdbba9frFj0=OqFfea0dXdd9vqai=hGuQ8kuc9pgc9s8qqaq=dirpe0xb9q8qiLsFr0=vr0=vr0dc8meaabaqaciaacaGaaeqabaqabeGadaaakeaaiiGacqWF0oazdaWgaaWcbaGaem4AaSgabeaakiabg2da9maalaaabaGaemOBa42aaSbaaSqaaiabgUcaRiabgUcaRiabdUgaRbqabaaakeaacqWGobGtdaaeqaqaaiab=17aUnaaBaaaleaacqWGPbqAaeqaaaqaaiabdMgaPjabdEgaNbqab0GaeyyeIuoakiabdcfaqnaaBaaaleaacqWGPbqAcqWGNbWzcqWGRbWAaeqaaOGaeiikaGIae8hUdeNaeiykaKcaaiabcYcaSiabbccaGiabdUgaRjabg2da9iabigdaXiabcYcaSiabc6caUiabc6caUiabc6caUiabcYcaSiabdUealjabcYcaSiaaxMaacaWLjaWaaeWaceaacqaIXaqmcqaI2aGnaiaawIcacaGLPaaaaaa@57A7@

where *n*_++*k *_= ∑_*ig *_*n*_*igk*_, and

νi=NiN∑gkPigk(θ)δk=NiNpr(D=i).
 MathType@MTEF@5@5@+=feaafiart1ev1aaatCvAUfKttLearuWrP9MDH5MBPbIqV92AaeXatLxBI9gBaebbnrfifHhDYfgasaacH8akY=wiFfYdH8Gipec8Eeeu0xXdbba9frFj0=OqFfea0dXdd9vqai=hGuQ8kuc9pgc9s8qqaq=dirpe0xb9q8qiLsFr0=vr0=vr0dc8meaabaqaciaacaGaaeqabaqabeGadaaakeaaiiGacqWF9oGBdaWgaaWcbaGaemyAaKgabeaakiabg2da9maalaaabaGaemOta40aaSbaaSqaaiabdMgaPbqabaaakeaacqWGobGtdaaeqaqaaiabdcfaqnaaBaaaleaacqWGPbqAcqWGNbWzcqWGRbWAaeqaaaqaaiabdEgaNjabdUgaRbqab0GaeyyeIuoakiabcIcaOiab=H7aXjabcMcaPiab=r7aKnaaBaaaleaacqWGRbWAaeqaaaaakiabg2da9maalaaabaGaemOta40aaSbaaSqaaiabdMgaPbqabaaakeaacqWGobGtcqqGWbaCcqqGYbGCcqGGOaakcqWGebarcqGH9aqpcqWGPbqAcqGGPaqkaaGaeiOla4caaa@534E@

Substituting the right side of (16) for *δ*_*k *_in *L*, the profile loglikelihood is obtained as, aside from additive constants,

LP=∑igknigklog⁡Pigk∗(θ),
 MathType@MTEF@5@5@+=feaafiart1ev1aaatCvAUfKttLearuWrP9MDH5MBPbIqV92AaeXatLxBI9gBaebbnrfifHhDYfgasaacH8akY=wiFfYdH8Gipec8Eeeu0xXdbba9frFj0=OqFfea0dXdd9vqai=hGuQ8kuc9pgc9s8qqaq=dirpe0xb9q8qiLsFr0=vr0=vr0dc8meaabaqaciaacaGaaeqabaqabeGadaaakeaacqWGmbatdaWgaaWcbaGaemiuaafabeaakiabg2da9maaqafabaGaemOBa42aaSbaaSqaaiabdMgaPjabdEgaNjabdUgaRbqabaaabaGaemyAaKMaem4zaCMaem4AaSgabeqdcqGHris5aOGagiiBaWMaei4Ba8Maei4zaCMaemiuaa1aa0baaSqaaiabdMgaPjabdEgaNjabdUgaRbqaaiabgEHiQaaakiabcIcaOGGaciab=H7aXjabcMcaPiabcYcaSaaa@4AD4@

where

Pigk∗(θ)=νiPigk(θ)∑igνiPigk(θ).
 MathType@MTEF@5@5@+=feaafiart1ev1aaatCvAUfKttLearuWrP9MDH5MBPbIqV92AaeXatLxBI9gBaebbnrfifHhDYfgasaacH8akY=wiFfYdH8Gipec8Eeeu0xXdbba9frFj0=OqFfea0dXdd9vqai=hGuQ8kuc9pgc9s8qqaq=dirpe0xb9q8qiLsFr0=vr0=vr0dc8meaabaqaciaacaGaaeqabaqabeGadaaakeaacqWGqbaudaqhaaWcbaGaemyAaKMaem4zaCMaem4AaSgabaGaey4fIOcaaOGaeiikaGccciGae8hUdeNaeiykaKIaeyypa0ZaaSaaaeaacqWF9oGBdaWgaaWcbaGaemyAaKgabeaakiabdcfaqnaaBaaaleaacqWGPbqAcqWGNbWzcqWGRbWAaeqaaOGaeiikaGIae8hUdeNaeiykaKcabaWaaabeaeaacqWF9oGBdaWgaaWcbaGaemyAaKgabeaaaeaacqWGPbqAcqWGNbWzaeqaniabggHiLdGccqWGqbaudaWgaaWcbaGaemyAaKMaem4zaCMaem4AaSgabeaakiabcIcaOiab=H7aXjabcMcaPaaacqGGUaGlaaa@552D@

It is easy to see that

Pigk∗Pi0k∗=PigkPi0k, for i=0,1,g=0,...,Q−1,k=1,...,K
 MathType@MTEF@5@5@+=feaafiart1ev1aaatCvAUfKttLearuWrP9MDH5MBPbIqV92AaeXatLxBI9gBaebbnrfifHhDYfgasaacH8akY=wiFfYdH8Gipec8Eeeu0xXdbba9frFj0=OqFfea0dXdd9vqai=hGuQ8kuc9pgc9s8qqaq=dirpe0xb9q8qiLsFr0=vr0=vr0dc8meaabaqaciaacaGaaeqabaqabeGadaaakeaadaWcaaqaaiabdcfaqnaaDaaaleaacqWGPbqAcqWGNbWzcqWGRbWAaeaacqGHxiIkaaaakeaacqWGqbaudaqhaaWcbaGaemyAaKMaeGimaaJaem4AaSgabaGaey4fIOcaaaaakiabg2da9maalaaabaGaemiuaa1aaSbaaSqaaiabdMgaPjabdEgaNjabdUgaRbqabaaakeaacqWGqbaudaWgaaWcbaGaemyAaKMaeGimaaJaem4AaSgabeaaaaGccqGGSaalcqqGGaaicqqGMbGzcqqGVbWBcqqGYbGCcqqGGaaicqWGPbqAcqGH9aqpcqaIWaamcqGGSaalcqaIXaqmcqGGSaalcqWGNbWzcqGH9aqpcqaIWaamcqGGSaalcqGGUaGlcqGGUaGlcqGGUaGlcqGGSaalcqWGrbqucqGHsislcqaIXaqmcqGGSaalcqWGRbWAcqGH9aqpcqaIXaqmcqGGSaalcqGGUaGlcqGGUaGlcqGGUaGlcqGGSaalcqWGlbWsaaa@65AF@

and

P10k∗P00k∗=ν1P10kν0P00k,k=1,...,K.
 MathType@MTEF@5@5@+=feaafiart1ev1aaatCvAUfKttLearuWrP9MDH5MBPbIqV92AaeXatLxBI9gBaebbnrfifHhDYfgasaacH8akY=wiFfYdH8Gipec8Eeeu0xXdbba9frFj0=OqFfea0dXdd9vqai=hGuQ8kuc9pgc9s8qqaq=dirpe0xb9q8qiLsFr0=vr0=vr0dc8meaabaqaciaacaGaaeqabaqabeGadaaakeaadaWcaaqaaiabdcfaqnaaDaaaleaacqaIXaqmcqaIWaamcqWGRbWAaeaacqGHxiIkaaaakeaacqWGqbaudaqhaaWcbaGaeGimaaJaeGimaaJaem4AaSgabaGaey4fIOcaaaaakiabg2da9maalaaabaGaeqyVd42aaSbaaSqaaiabigdaXaqabaGccqWGqbaudaWgaaWcbaGaeGymaeJaeGimaaJaem4AaSgabeaaaOqaaiabe27aUnaaBaaaleaacqaIWaamaeqaaOGaemiuaa1aaSbaaSqaaiabicdaWiabicdaWiabdUgaRbqabaaaaOGaeiilaWIaem4AaSMaeyypa0JaeGymaeJaeiilaWIaeiOla4IaeiOla4IaeiOla4IaeiilaWIaem4saSKaeiOla4caaa@527C@

From (9) we thus have

Pigk∗(θ)=∑h∈S(G=g)exp⁡{Λih(X;θ∗)}∑i=01∑h=0M−1exp⁡{Λih(X;θ∗)},
 MathType@MTEF@5@5@+=feaafiart1ev1aaatCvAUfKttLearuWrP9MDH5MBPbIqV92AaeXatLxBI9gBamrtHrhAL1wy0L2yHvtyaeHbnfgDOvwBHrxAJfwnaebbnrfifHhDYfgasaacH8akY=wiFfYdH8Gipec8Eeeu0xXdbba9frFj0=OqFfea0dXdd9vqai=hGuQ8kuc9pgc9s8qqaq=dirpe0xb9q8qiLsFr0=vr0=vr0dc8meaabaqaciaacaGaaeqabaWaaeGaeaaakeaacqWGqbaudaqhaaWcbaGaemyAaKMaem4zaCMaem4AaSgabaGaey4fIOcaaOGaeiikaGccciGae8hUdeNaeiykaKIaeyypa0ZaaSaaaeaadaaeqaqaaiGbcwgaLjabcIha4jabcchaWbWcbaGaemiAaGMaeyicI4mcdaGae4NeXpLaeiikaGIaem4raCKaeyypa0Jaem4zaCMaeiykaKcabeqdcqGHris5aOWaaiWaaeaacqqHBoatdaWgaaWcbaGaemyAaKMaemiAaGgabeaakiabcIcaOiabdIfayjabcUda7iab=H7aXnaaCaaaleqabaGaey4fIOcaaOGaeiykaKcacaGL7bGaayzFaaaabaWaaabmaeaadaaeWaqaaiGbcwgaLjabcIha4jabcchaWbWcbaGaemiAaGMaeyypa0JaeGimaadabaGaemyta0KaeyOeI0IaeGymaedaniabggHiLdGcdaGadaqaaiabfU5amnaaBaaaleaacqWGPbqAcqWGObaAaeqaaOGaeiikaGIaemiwaGLaei4oaSJae8hUde3aaWbaaSqabeaacqGHxiIkaaGccqGGPaqkaiaawUhacaGL9baaaSqaaiabdMgaPjabg2da9iabicdaWaqaaiabigdaXaqdcqGHris5aaaakiabcYcaSaaa@7FA5@

where

Λ_*ih*_(*X*; θ^*^) = *i*(α^* ^+ *β*_*h *_+ *X*'*β*_*X *_+ *X*'*β*_*hX*_) + *m*(*h*, *X*; *γ*),

and α^* ^= *α *+ log(*ν*_1_/*ν*_0_).

### Appendix 3 expressions of complete-data score and information matrix

Simple algebra leads to

ΨuC(θ∗)=∂∂θ∗log⁡Pr⁡∗(Du,Hu|Xu;θ∗)=∂ΛDuHu(Xu;θ∗)∂θ∗−∑i′h′exp⁡{Λi′h′(Xu;θ∗}∑i″h″exp⁡{Λi″h″(Xu;θ∗}∂Λi′h′(Xu;θ∗)∂θ∗=∂ΛDuHu∗(Xu;θ∗)∂θ∗−E∗{∂ΛDH(X;θ∗)∂θ∗|X=Xu},
 MathType@MTEF@5@5@+=feaafiart1ev1aaatCvAUfKttLearuWrP9MDH5MBPbIqV92AaeXatLxBI9gBaebbnrfifHhDYfgasaacH8akY=wiFfYdH8Gipec8Eeeu0xXdbba9frFj0=OqFfea0dXdd9vqai=hGuQ8kuc9pgc9s8qqaq=dirpe0xb9q8qiLsFr0=vr0=vr0dc8meaabaqaciaacaGaaeqabaqabeGadaaakeaafaqadeWabaaabaGaeuiQdK1aa0baaSqaaiabdwha1bqaaiabdoeadbaakiabcIcaOGGaciab=H7aXnaaCaaaleqabaGaey4fIOcaaOGaeiykaKIaeyypa0ZaaSaaaeaacqGHciITaeaacqGHciITcqWF4oqCdaahaaWcbeqaaiabgEHiQaaaaaGccyGGSbaBcqGGVbWBcqGGNbWzcyGGqbaucqGGYbGCdaahaaWcbeqaaiabgEHiQaaakiabcIcaOiabdseaenaaBaaaleaacqWG1bqDaeqaaOGaeiilaWIaemisaG0aaSbaaSqaaiabdwha1bqabaGccqGG8baFcqWGybawdaWgaaWcbaGaemyDauhabeaakiabcUda7iab=H7aXnaaCaaaleqabaGaey4fIOcaaOGaeiykaKcabaGaeyypa0ZaaSaaaeaacqGHciITcqqHBoatdaWgaaWcbaGaemiraq0aaSbaaWqaaiabdwha1bqabaWccqWGibasdaWgaaadbaGaemyDauhabeaaaSqabaGccqGGOaakcqWGybawdaWgaaWcbaGaemyDauhabeaakiabcUda7iab=H7aXnaaCaaaleqabaGaey4fIOcaaOGaeiykaKcabaGaeyOaIyRae8hUde3aaWbaaSqabeaacqGHxiIkaaaaaOGaeyOeI0YaaabuaeaadaWcaaqaaiGbcwgaLjabcIha4jabcchaWnaacmqabaGaeu4MdW0aaSbaaSqaaiqbdMgaPzaafaGafmiAaGMbauaaaeqaaOGaeiikaGIaemiwaG1aaSbaaSqaaiabdwha1bqabaGccqGG7aWocqWF4oqCdaahaaWcbeqaaiabgEHiQaaaaOGaay5Eaiaaw2haaaqaamaaqababaGagiyzauMaeiiEaGNaeiiCaa3aaiWabeaacqqHBoatdaWgaaWcbaGafmyAaKMbayaacuWGObaAgaGbaaqabaGccqGGOaakcqWGybawdaWgaaWcbaGaemyDauhabeaakiabcUda7iab=H7aXnaaCaaaleqabaGaey4fIOcaaaGccaGL7bGaayzFaaaaleaacuWGPbqAgaGbaiqbdIgaOzaagaaabeqdcqGHris5aaaakmaalaaabaGaeyOaIyRaeu4MdW0aaSbaaSqaaiqbdMgaPzaafaGafmiAaGMbauaaaeqaaOGaeiikaGIaemiwaG1aaSbaaSqaaiabdwha1bqabaGccqGG7aWocqWF4oqCdaahaaWcbeqaaiabgEHiQaaakiabcMcaPaqaaiabgkGi2kab=H7aXnaaCaaaleqabaGaey4fIOcaaaaaaeaacuWGPbqAgaqbaiqbdIgaOzaafaaabeqdcqGHris5aaGcbaGaeyypa0ZaaSaaaeaacqGHciITcqqHBoatdaqhaaWcbaGaemiraq0aaSbaaWqaaiabdwha1bqabaWccqWGibasdaWgaaadbaGaemyDauhabeaaaSqaaiabgEHiQaaakiabcIcaOiabdIfaynaaBaaaleaacqWG1bqDaeqaaOGaei4oaSJae8hUde3aaWbaaSqabeaacqGHxiIkaaGccqGGPaqkaeaacqGHciITcqWF4oqCdaahaaWcbeqaaiabgEHiQaaaaaGccqGHsislcqWGfbqrdaahaaWcbeqaaiabgEHiQaaakmaacmqabaWaaqGaceaadaWcaaqaaiabgkGi2kabfU5amnaaBaaaleaacqWGebarcqWGibasaeqaaOGaeiikaGIaemiwaGLaei4oaSJae8hUde3aaWbaaSqabeaacqGHxiIkaaGccqGGPaqkaeaacqGHciITcqWF4oqCdaahaaWcbeqaaiabgEHiQaaaaaaakiaawIa7aiabdIfayjabg2da9iabdIfaynaaBaaaleaacqWG1bqDaeqaaaGccaGL7bGaayzFaaGaeiilaWcaaaaa@E13E@

where E^*^(·|*X*) denotes expectation with respect to Pr^*^(*D*, *H*|*X*; θ^*^).

The negative derivative of ΨuC
 MathType@MTEF@5@5@+=feaafiart1ev1aaatCvAUfKttLearuWrP9MDH5MBPbIqV92AaeXatLxBI9gBaebbnrfifHhDYfgasaacH8akY=wiFfYdH8Gipec8Eeeu0xXdbba9frFj0=OqFfea0dXdd9vqai=hGuQ8kuc9pgc9s8qqaq=dirpe0xb9q8qiLsFr0=vr0=vr0dc8meaabaqaciaacaGaaeqabaqabeGadaaakeaacqqHOoqwdaqhaaWcbaGaemyDauhabaGaem4qameaaaaa@30EA@ (θ^*^) is given by

−∂∂θ∗ΨuC(θ∗)=−[∂ΛDuHu(Xu;θ∗)∂θ∗∂θ∗′−E∗{∂ΛDH(Xu;θ∗)∂θ∗∂θ∗′|X=Xu}]+∑D,H∂ΛDH(Xu;θ∗)∂θ∗∂∂θ∗Pr⁡∗(D,H|Xu;θ∗)
 MathType@MTEF@5@5@+=feaafiart1ev1aaatCvAUfKttLearuWrP9MDH5MBPbIqV92AaeXatLxBI9gBaebbnrfifHhDYfgasaacH8akY=wiFfYdH8Gipec8Eeeu0xXdbba9frFj0=OqFfea0dXdd9vqai=hGuQ8kuc9pgc9s8qqaq=dirpe0xb9q8qiLsFr0=vr0=vr0dc8meaabaqaciaacaGaaeqabaqabeGadaaakeaafaqabeGabaaabaGaeyOeI0YaaSaaaeaacqGHciITaeaacqGHciITiiGacqWF4oqCdaahaaWcbeqaaiabgEHiQaaaaaGccqqHOoqwdaqhaaWcbaGaemyDauhabaGaem4qameaaOGaeiikaGIae8hUde3aaWbaaSqabeaacqGHxiIkaaGccqGGPaqkcqGH9aqpcqGHsisldaWadiqaamaalaaabaGaeyOaIyRaeu4MdW0aaSbaaSqaaiabdseaenaaBaaameaacqWG1bqDaeqaaSGaemisaG0aaSbaaWqaaiabdwha1bqabaaaleqaaOGaeiikaGIaemiwaG1aaSbaaSqaaiabdwha1bqabaGccqGG7aWocqWF4oqCdaahaaWcbeqaaiabgEHiQaaakiabcMcaPaqaaiabgkGi2kab=H7aXnaaCaaaleqabaGaey4fIOcaaOGaeyOaIyRae8hUde3aaWbaaSqabeaacuGHxiIkgaqbaaaaaaGccqGHsislcqWGfbqrdaahaaWcbeqaaiabgEHiQaaakmaacmqabaWaaSaaaeaacqGHciITcqqHBoatdaWgaaWcbaGaemiraqKaemisaGeabeaakiabcIcaOiabdIfaynaaBaaaleaacqWG1bqDaeqaaOGaei4oaSJae8hUde3aaWbaaSqabeaacqGHxiIkaaGccqGGPaqkaeaacqGHciITcqWF4oqCdaahaaWcbeqaaiabgEHiQaaakiabgkGi2kab=H7aXnaaCaaaleqabaGafy4fIOIbauaaaaaaaOGaeiiFaWNaemiwaGLaeyypa0JaemiwaG1aaSbaaSqaaiabdwha1bqabaaakiaawUhacaGL9baaaiaawUfacaGLDbaaaeaacqGHRaWkdaaeqbqaamaalaaabaGaeyOaIyRaeu4MdW0aaSbaaSqaaiabdseaejabdIeaibqabaGccqGGOaakcqWGybawdaWgaaWcbaGaemyDauhabeaakiabcUda7iab=H7aXnaaCaaaleqabaGaey4fIOcaaOGaeiykaKcabaGaeyOaIyRae8hUde3aaWbaaSqabeaacqGHxiIkaaaaaOWaaSaaaeaacqGHciITaeaacqGHciITcqWF4oqCdaahaaWcbeqaaiabgEHiQaaaaaaabaGaemiraqKaeiilaWIaemisaGeabeqdcqGHris5aOGagiiuaaLaeiOCai3aaWbaaSqabeaacqGHxiIkaaGccqGGOaakcqWGebarcqGGSaalcqWGibascqGG8baFcqWGybawdaWgaaWcbaGaemyDauhabeaakiabcUda7iab=H7aXnaaCaaaleqabaGaey4fIOcaaOGaeiykaKcaaaaa@AA37@

Note that the bracket term has mean zero. Hence the complete-data information matrix can be approximated by

∑D,H∂ΛDH(Xu;θ∗)∂θ∗∂∂θ∗Pr⁡∗(D,H|Xu:θ∗)=∑D,H∂ΛDH(Xu;θ∗)∂θ∗∂log⁡Pr⁡∗(D,H|Xu;θ∗)∂θ∗Pr⁡∗(D,H|Xu;θ∗)=V∗{∂ΛDH(X;θ∗)∂θ∗|X=Xu},
 MathType@MTEF@5@5@+=feaafiart1ev1aaatCvAUfKttLearuWrP9MDH5MBPbIqV92AaeXatLxBI9gBaebbnrfifHhDYfgasaacH8akY=wiFfYdH8Gipec8Eeeu0xXdbba9frFj0=OqFfea0dXdd9vqai=hGuQ8kuc9pgc9s8qqaq=dirpe0xb9q8qiLsFr0=vr0=vr0dc8meaabaqaciaacaGaaeqabaqabeGadaaakeaafaqaaeWacaaabaaabaWaaabuaeaadaWcaaqaaiabgkGi2kabfU5amnaaBaaaleaacqWGebarcqWGibasaeqaaOGaeiikaGIaemiwaG1aaSbaaSqaaiabdwha1bqabaGccqGG7aWoiiGacqWF4oqCdaahaaWcbeqaaiabgEHiQaaakiabcMcaPaqaaiabgkGi2kab=H7aXnaaCaaaleqabaGaey4fIOcaaaaaaeaacqWGebarcqGGSaalcqWGibasaeqaniabggHiLdGcdaWcaaqaaiabgkGi2cqaaiabgkGi2kab=H7aXnaaCaaaleqabaGaey4fIOcaaaaakiGbccfaqjabckhaYnaaCaaaleqabaGaey4fIOcaaOGaeiikaGIaemiraqKaeiilaWIaemisaGKaeiiFaWNaemiwaG1aaSbaaSqaaiabdwha1bqabaGccqGG6aGocqWF4oqCdaahaaWcbeqaaiabgEHiQaaakiabcMcaPaqaaiabg2da9aqaamaaqafabaWaaSaaaeaacqGHciITcqqHBoatdaWgaaWcbaGaemiraqKaemisaGeabeaakiabcIcaOiabdIfaynaaBaaaleaacqWG1bqDaeqaaOGaei4oaSJae8hUde3aaWbaaSqabeaacqGHxiIkaaGccqGGPaqkaeaacqGHciITcqWF4oqCdaahaaWcbeqaaiabgEHiQaaaaaaabaGaemiraqKaeiilaWIaemisaGeabeqdcqGHris5aOWaaSaaaeaacqGHciITcyGGSbaBcqGGVbWBcqGGNbWzcyGGqbaucqGGYbGCdaahaaWcbeqaaiabgEHiQaaakiabcIcaOiabdseaejabcYcaSiabdIeaijabcYha8jabdIfaynaaBaaaleaacqWG1bqDaeqaaOGaei4oaSJae8hUde3aaWbaaSqabeaacqGHxiIkaaGccqGGPaqkaeaacqGHciITcqWF4oqCdaahaaWcbeqaaiabgEHiQaaaaaGccyGGqbaucqGGYbGCdaahaaWcbeqaaiabgEHiQaaakiabcIcaOiabdseaejabcYcaSiabdIeaijabcYha8jabdIfaynaaBaaaleaacqWG1bqDaeqaaOGaei4oaSJae8hUde3aaWbaaSqabeaacqGHxiIkaaGccqGGPaqkaeaacqGH9aqpaeaacqWGwbGvdaahaaWcbeqaaiabgEHiQaaakmaacmqabaWaaSaaaeaacqGHciITcqqHBoatdaWgaaWcbaGaemiraqKaemisaGeabeaakiabcIcaOiabdIfayjabcUda7iab=H7aXnaaCaaaleqabaGaey4fIOcaaOGaeiykaKcabaGaeyOaIyRae8hUde3aaWbaaSqabeaacqGHxiIkaaaaaOGaeiiFaWNaemiwaGLaeyypa0JaemiwaG1aaSbaaSqaaiabdwha1bqabaaakiaawUhacaGL9baacqGGSaalaaaaaa@BAE1@

where V^*^(·|*X*) denotes the variance with respect to Pr^*^(*D*, *H*|*X*; θ^*^). The negative derivative, of ∑_*u *_∑_*h *_*ρ*_*hu*_ΨuC
 MathType@MTEF@5@5@+=feaafiart1ev1aaatCvAUfKttLearuWrP9MDH5MBPbIqV92AaeXatLxBI9gBaebbnrfifHhDYfgasaacH8akY=wiFfYdH8Gipec8Eeeu0xXdbba9frFj0=OqFfea0dXdd9vqai=hGuQ8kuc9pgc9s8qqaq=dirpe0xb9q8qiLsFr0=vr0=vr0dc8meaabaqaciaacaGaaeqabaqabeGadaaakeaacqqHOoqwdaqhaaWcbaGaemyDauhabaGaem4qameaaaaa@30EA@(θ^*^) can thus be approximated by ∑_*u *_∑_*h *_*ρ*_*hu*_v^* ^(*X*_*u*_; θ^*^) = ∑_*u*_v^* ^(*X*_*u*_; θ^*^), where

v∗(Xu)=V∗{∂ΛDH(X;θ∗)∂θ∗|X=Xu}.
 MathType@MTEF@5@5@+=feaafiart1ev1aaatCvAUfKttLearuWrP9MDH5MBPbIqV92AaeXatLxBI9gBaebbnrfifHhDYfgasaacH8akY=wiFfYdH8Gipec8Eeeu0xXdbba9frFj0=OqFfea0dXdd9vqai=hGuQ8kuc9pgc9s8qqaq=dirpe0xb9q8qiLsFr0=vr0=vr0dc8meaabaqaciaacaGaaeqabaqabeGadaaakeaacqWG2bGDdaahaaWcbeqaaiabgEHiQaaakiabcIcaOiabdIfaynaaBaaaleaacqWG1bqDaeqaaOGaeiykaKIaeyypa0JaemOvay1aaWbaaSqabeaacqGHxiIkaaGcdaGadeqaamaalaaabaGaeyOaIyRaeu4MdW0aaSbaaSqaaiabdseaejabdIeaibqabaGccqGGOaakcqWGybawcqGG7aWoiiGacqWF4oqCdaahaaWcbeqaaiabgEHiQaaakiabcMcaPaqaaiabgkGi2kab=H7aXnaaCaaaleqabaGaey4fIOcaaaaakiabcYha8jabdIfayjabg2da9iabdIfaynaaBaaaleaacqWG1bqDaeqaaaGccaGL7bGaayzFaaGaeiOla4caaa@5147@

## References

[B1] Schaid DJ (2004). Evaluating associations of haplotypes with traits. Genet Epidemiol.

[B2] Prentice RL, Pyke R (1979). Logistic disease incidence models and case-control studies. Biometrika.

[B3] Excoffier L, Slatkin M (1995). Maximum-likelihood estimation of molecular haplotype frequencies in a diploid populaton. Mol Biol Evol.

[B4] Li SS, Khalid N, Carlson C, Zhao LP (2003). Estimating haplotype frequencies and standard errors for multiple single nucleotide polymorphisms. Biostatistics.

[B5] Stephens M, Smith NJ, Donnelly P (2001). A new statistical method for haplotype reconstruction from population data. Am J Hum Genet.

[B6] Niu T, Qin Z, Xu X, Lin J (2002). Bayesian haplotype inference for multiple linked single-nucleotide polymorphisims. Am J Hum Genet.

[B7] Epstein MP, Satten GA (2003). Inference on haplotype effects in case-control studies using unphased genotype data. Am J Hum Genet.

[B8] Chatterjee N, Carroll RJ (2005). Semiparametric maximum likelihood estimation in case-control studies of gene-environment interactions. Biometrika.

[B9] Spinka C, Carroll RJ, Chatterjee N (2005). Analysis of case-control studies of genetic and environmental factors with missing genetic information and haplotype-phase ambiguity. Genet Epidemiol.

[B10] Agresti A (2002). Categorical data analysis.

[B11] Hosmer DW, Lemeshow S (2000). Applied logistic regression.

[B12] Zhao LP, Li SS, Khalid N (2003). A method for the assessment of disease associations with single-nucleotide polymorphism haplotypes and environmental variables in case-control studies. Am J Hum Genet.

[B13] Hartl DL (1988). A primer of population genetics.

[B14] Satten GA, Epstein MP (2004). Comparison of prospective and retrospective methods for haplotype inference in case-control studies. Genet Epidemiol.

[B15] Scott AJ, Wild CJ (1997). Fitting regression models to case-control data by maximum likelihood. Biometrika.

[B16] Louis TA (1982). Finding the observed information matrix when using the EM algorithm. J R Statist Soc B.

[B17] Kao JT, Wen HC, Chien KL, Hsu HC, Lin SW (2003). A novel genetic variant in the apolipoprotein A5 gene is associated with hypertriglyceridemia. Hum Mol Genet.

[B18] Tzeng JY, Wang CH, Kao JT, Hsiao CK (2006). Regression-based association analyis with clustered haplotypes through use of genotypes. Am J Hum Genet.

[B19] Pennacchio LA, Oliver M, Hubacek JA, Cohen JC, Cox DR, Fruchart JC, Krauss RM, Rubin EM (2001). An apolipoprotein influencing triglycerides in humans and mice revealed by comparative sequencing. Science.

[B20] Stram DO, Pearce L, Henderson BE, Thomas DC (2003). Modeling and E-M estimation of haplotype-specific relative risks from genotype data for a case-control study of unrelated individuals. Hum Hered.

[B21] Lake SL, Lyon H, Tantisira K, Silverman EK, Weiss ST, Laird NM, Schaid DJ (2003). Estimation and tests of haplotype-environment interaction when linkage phase in ambiguous. Hum Hered.

[B22] Cordell HJ, Clayton DG (2002). A unified stepwise regression procedure for evaluating the relative effects of polymorphisms within a gene using case/control or family data: application to HLA in type 1 diabetes. Am J Hum Genet.

[B23] Tibshirani R (1996). Regression shrinkage and selection via the Lasso. J R Stat Soc B.

[B24] Devlin B, Roeder K, Wasserman L (2003). Analysis of multilocus models of association. Genet Epidemiol.

[B25] Benjamini Y, Hochberg Y (1995). Controlling the false discovery rate: a practical and powerful approach to multiple testing. J R Stat Soc B.

